# Phenotypic variation in photosynthetic traits in wheat grown under field versus glasshouse conditions

**DOI:** 10.1093/jxb/erac096

**Published:** 2022-03-10

**Authors:** Cristina R G Sales, Gemma Molero, John R Evans, Samuel H Taylor, Ryan Joynson, Robert T Furbank, Anthony Hall, Elizabete Carmo-Silva

**Affiliations:** 1 Lancaster Environment Centre, Lancaster University, Library Avenue, Lancaster LA1 4YQ, UK; 2 Department of Plant Sciences, University of Cambridge, Downing Street, Cambridge CB2 3EA, UK; 3 International Maize and Wheat Improvement Centre (CIMMYT), Int. Apdo. Postal 6-641, 06600 Mexico, DF, Mexico; 4 KWS Momont Recherche, 7 rue de Martinval, 59246 Mons-en-Pévèle, France; 5 ARC Centre of Excellence for Translational Photosynthesis, Research School of Biology, The Australian National University, Canberra ACT 2601, Australia; 6 Organisms and Ecosystems, Earlham Institute, Norwich Research Park, Norwich NR4 7UG, UK; 7 Limagrain Europe, CS 3911, 63720 Chappes, France; 8 University of Essex, UK

**Keywords:** Field, glasshouse, hyperspectral reflectance, photosynthesis, Rubisco, *Triticum aestivum*

## Abstract

Recognition of the untapped potential of photosynthesis to improve crop yields has spurred research to identify targets for breeding. The CO_2_-fixing enzyme Rubisco is characterized by a number of inefficiencies, and frequently limits carbon assimilation at the top of the canopy, representing a clear target for wheat improvement. Two bread wheat lines with similar genetic backgrounds and contrasting *in vivo* maximum carboxylation activity of Rubisco per unit leaf nitrogen (*V*_c,max,25_/*N*_area_) determined using high-throughput phenotyping methods were selected for detailed study from a panel of 80 spring wheat lines. Detailed phenotyping of photosynthetic traits in the two lines using glasshouse-grown plants showed no difference in *V*_c,max,25_/*N*_area_ determined directly via *in vivo* and *in vitro* methods. Detailed phenotyping of glasshouse-grown plants of the 80 wheat lines also showed no correlation between photosynthetic traits measured via high-throughput phenotyping of field-grown plants. Our findings suggest that the complex interplay between traits determining crop productivity and the dynamic environments experienced by field-grown plants needs to be considered in designing strategies for effective wheat crop yield improvement when breeding for particular environments.

## Teaser

Wheat plants grown in the glasshouse show different physiological properties compared with plants grown under dynamic field conditions, highlighting the need to consider realistic environmental conditions when breeding for particular environments.

## Introduction

Global food demand is expected to double in the next 50 years or so due to the growing world population and dietary changes (Tilman and Clark, 2015). Wheat alone provides >20% of the calories and the protein for the world’s population (Braun *et al.*, 2010), and theoretical analyses estimate that genetic gains in wheat would have to increase at a rate of 2.4% per year to meet predicted global demand (Hawkesford *et al.*, 2013; Ray *et al.*, 2013). Past genetic gains in bread wheat have largely resulted from improvements in harvest index (HI) rather than increased biomass. Further large increases in HI are unlikely, but an opportunity exists for increasing biomass production and harvestable grain (Parry *et al.*, 2011; Fischer *et al.*, 2014; Furbank *et al.*, 2020).

Photosynthesis is the primary determinant of biomass production. The maximum theoretical efficiency with which the sun’s energy can be captured as biomass by C_3_ plants is ~4.6% (Zhu *et al.*, 2008), although it rarely exceeds a third of this value in wheat under field conditions (Parry *et al.*, 2011). Improving conversion efficiency is a thriving area of research, with potential to significantly increase crop yields (Long *et al.*, 2006; Zhu *et al.*, 2010; Parry *et al.*, 2011; Driever *et al.*, 2017; Yadav *et al.*, 2018; Simkin *et al.*, 2019). To investigate whether these attributes can be improved via breeding, the presence of existing genetic variation in a species germplasm is a prerequisite. Genetic variation in photosynthesis has been reported in wheat (Driever *et al.*, 2014; Gaju *et al.*, 2016; Carmo-Silva *et al.*, 2017; Pennacchi *et al.*, 2018; Molero *et al.*, 2019; Silva-Pérez *et al*., 2020). Despite plant primary production being dependent on photosynthesis, a positive correlation between photosynthetic rates and yield is not always found (Murthy and Singh, 1979; Evans, 1983, Sadras *et al.*, 2012; Driever *et al.*, 2014). When considering yield increases achieved over the last century, one explanation for this lack of correlation is the dramatic impact of Green Revolution plant breeding strategies that increased allocation of primary production into yield components (reviewed by Gifford and Evans, 1981), a strategy that has been predicted to now be reaching its natural limit (Zhu *et al.*, 2010). Nonetheless, some studies have found positive correlations between flag leaf photosynthetic rates and grain yield in wheat (Gaju *et al.*, 2016; Carmo-Silva *et al.*, 2017), but processes underlying the observed variation in photosynthesis and how it relates to yield warrant further study (Flood *et al.*, 2011; Lawson *et al.*, 2012).

It is well known that plant performance is highly affected by environmental conditions. Experiments under controlled or glasshouse conditions are often performed aiming to assess genetic yield potential; however, translation between results obtained under field and controlled conditions is challenging (reviewed by Poorter *et al.*, 2016), with some studies showing similar physiological responses across experiments (Lovell *et al.*, 2016) and others showing contrasting findings (Patterson *et al.*, 1977; Silva-Pérez *et al*., 2020). The wheat photosynthetic tails (PStails) panel is a rich resource to aid in understanding the underlying processes that determine variation in CO_2_ assimilation rates in wheat. The PStails panel is composed of 80 bread spring wheat lines (*Triticum aestivum* L.) assembled after screening a range of elite International Maize and Wheat Improvement Center (CIMMYT) spring wheat germplasm (Molero *et al*., 2017, 2019). The selection was based on lines contrasting for radiation use efficiency (RUE) at different growth stages, *in vivo* maximum carboxylation activity of Rubisco (*V*_c,max_), and respiration. After phenotyping photosynthetic traits in this germplasm in the field, two lines that are genetically similar but contrasting for *V*_c,max_ per unit leaf nitrogen, yield, and biomass at physiological maturity, were selected and further characterized in glasshouse conditions.

The present study focused on establishing the extent of photosynthetic diversity across the PStails panel and characterizing the two selected lines in detail. The initial aims of this study were to (i) identify lines in the PStails panel with contrasting photosynthetic traits but similar genetic background under field conditions; and (ii) establish the photosynthetic properties of the two contrasting lines through detailed phenotyping under glasshouse conditions. The lack of correspondence between most of the physiological properties displayed by the two genotypes under a field versus a semi-controlled environment led to a third objective: (iii) to evaluate the correlation for photosynthetic- and yield-related traits determined under glasshouse versus field conditions across the PStails panel. The findings support the need to carefully define aims and design experiments, given the lack of correlation between traits determined in plants of the wheat PStails panel grown under field versus glasshouse conditions.

## Materials and methods

### PStails panel: field conditions—plant material and growth

**Fig. 1.**
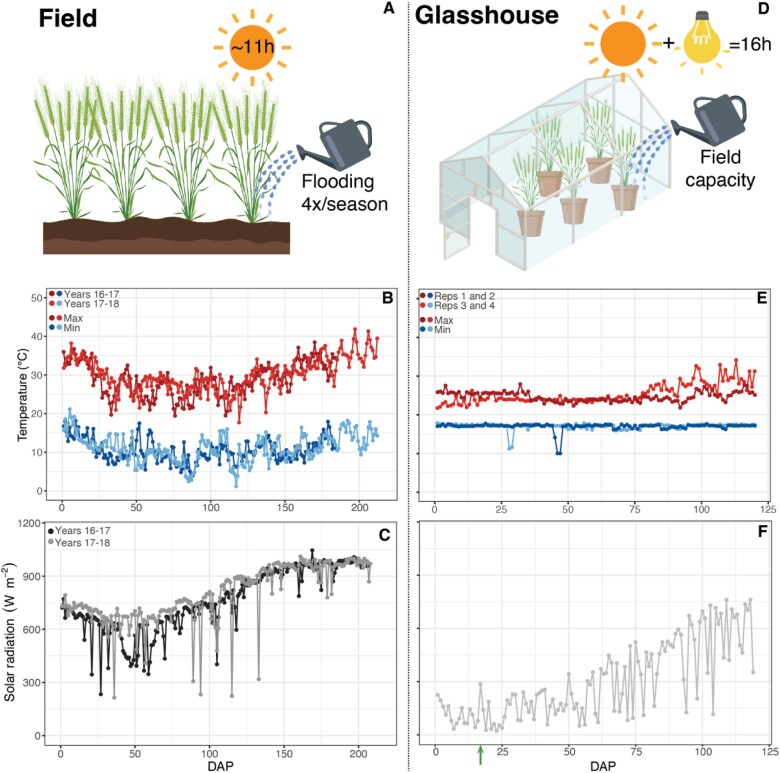
Schematic description and meteorology from the (A–C) field and (D–F) glasshouse experiments performed with the 80 wheat lines of the photosynthetic tails (PStails) panel; (B, E) daily maximum and minimum air temperature, and (C, F) maximum solar radiation during the experiments. Weather data for the field experiments are from December 2016 to May 2017 (Years 16–17), and from December 2017 to May 2018 (Years 17–18), from the weather station (http://www.siafeson.com/remas/index.php) located ~2 km from CIMMYT Experimental Station Norman E. Borlaug (CENEB). Temperature data for the glasshouse experiments are from sensors located inside the glasshouse; solar radiation is from the weather station (http://es-websupp.lancs.ac.uk/hazelrigg/) located ~1 km from Lancaster University, from December 2017 to March 2018. Days after planting (DAP) in (E) and (F) are shown for the first experimental block; the second block was sown at 17 DAP (green arrow).

The PStails panel is composed of 80 bread wheat lines (*T. aestivum* L.) selected from 150 lines of the High Biomass Association Panel (HiBAP; Molero *et al.*, 2019) and from 370 lines of the Bread Wheat Diversity Panel (Molero *et al.*, 2017; Supplementary Table S1), based on genetic diversity identified with genetic analysis and lines contrasting for RUE at different growth stages, *V*_c,max_, and respiration. The panel was evaluated in the field for 2 years (2016–2017 and 2017–2018) under fully irrigated conditions at the International Wheat Yield Partnership Phenotyping Platform (IWYP-Hub) situated at the International Maize and Wheat Improvement Centre (CIMMYT) Experimental Station Norman E. Borlaug (CENEB) in the Yaqui Valley, near Ciudad Obregon, Sonora, Mexico (27°24ʹN, 109°56ʹW, 38 masl). Maximum and minimum temperature, and maximum solar radiation (W m^–2^) during the 2 year field experiments (Fig. 1B, C) are from the weather station located ~2 km from the experimental station (http://www.siafeson.com/remas/index.php). Experimental design was an alpha-lattice with two replications in raised beds (two beds per plot, 0.8 m wide) with two rows per bed (0.24 m between rows) and 4 m long. Seeding rates were 102 kg ha^−1^. Appropriate weed disease and pest control were implemented to avoid yield limitations. Plots were fertilized with 50 kg N ha^−1^ (urea) and 50 kg P ha^−1^ at soil preparation, 50 kg N ha^−1^ with the first irrigation, and another 150 kg N ha^−1^ with the second irrigation.

### PStails panel: field conditions—hyperspectral reflectance measurements and SPAD

The full PStails panel was screened under field conditions using hyperspectral reflectance. Flag leaves were measured between 11.00 h and 14.00 h at booting stage [Zadoks stage (Zadoks *et al.*, 1974) between 4.3 and 4.5], anthesis (Zadoks 6.5), and grain filling (7 d after anthesis) using the protocol described by Silva-Perez *et al.* (2018). A FieldSpec®3 (Analytical Spectral Devices, Boulder, CO, USA) full-range spectroradiometer (350–2500 nm) was coupled via a fibre optic cable to a leaf. A mask was used to reduce the leaf-clip aperture, and a black circular gasket was pasted to the mask to avoid leaf damage and to eliminate potential entry of external light through the edges. One reflectance measurement was made per leaf lamina, and two measurements per plot measuring a total of two plots per entry. Leaf nitrogen content per unit leaf area (*N*_area_), leaf nitrogen content per unit dry mass (*N*_mass_), *V*_c,max,25_ per unit leaf nitrogen [*V*_c,max,25(HS)_/*N*_area_], electron transport rate [*J*_(HS)_], and SPAD (indication for chlorophyll content) were calculated as described in Silva-Perez *et al.* (2018).

### PStails panel: field conditions—photosynthetic measurements

Flag leaf photosynthetic rate was measured as carbon uptake using a LI-6400XT portable infrared gas analyser (IRGA) system (LI-COR, Lincoln, NE, USA) approximately at booting stage (Zadoks stage 4.3–4.5). The flag leaf net CO_2_ assimilation rate (*A*_CO2_) was estimated at a photosynthetically active radiation (PAR) of 1800 μmol m^–2^ s^–1^, air CO_2_ concentration in the reference analyser (CO_2__r) of 40 Pa, 300 µmol s^–1^ flow rate, and block temperature of 25 °C (here called *A*_sat_ as it was under saturating light). The average value of leaf vapour pressure deficit (VPD_leaf_) inside the chamber was 1.2 kPa across years.

### PStails—field conditions: phenology and yield components

Phenology of the plots was recorded at initiation of booting (Zadoks stage 4.5), heading (Zadoks stage 5.5), anthesis (Zadoks stage 6.5), and at physiological maturity (Zadoks stage 8.7) when 50% of the plants reached the phenological stage, as described by Pask *et al*. (2012). Plant height was measured as the length of five individual shoots per plot from the soil surface to the tip of the spike, excluding the awns.

At physiological maturity, determination of grain yield (GY) and yield components was conducted using standard protocols (Pask *et al*., 2012). A sample of 50 fertile shoots was taken from the area of the plot harvested to estimate yield components. The sample was oven-dried, weighed, and threshed to allow calculation of the HI, biomass at physiological maturity, thousand grain weight (TGW), and grains per square metre (GM2). GY was determined on a minimum of 4 m^2^. To avoid edge effects arising from extra solar radiation reaching border plants, under yield potential conditions, 50 cm of the plot edges were discarded before harvesting. From the harvest of each plot, a subsample of grains was weighed before and after drying (oven-dried to constant weight at 70 °C for 48 h) and the ratio of dry to fresh weight was used to determine dry GY and TGW. GM2 was calculated as [(GY/TGW)×1000]. Total biomass at physiological maturity was calculated from GY/HI.

### PStails panel: field conditions—DNA extraction and genotyping

Plant material was obtained from five plants per panel accession from field trials conducted in the CIMMYT field station in Ciuidad Obregon, Mexico. DNA was subsequently extracted from flag leaf material using a standard Qiagen DNeasy miniprep kit following the manufacturer’s protocols. Extracted DNA integrity and purity were determined using a Nanodrop2000 and quantified using the Qubit HS assay kit. All members of the PStails panel were subjected to enrichment capture sequencing using a custom MyBaits 12 Mbp, 120 bp RNA probe set based on the capture used by Gardiner *et al.* (2018) and Joynson *et al.* (2021). Enrichment capture was performed with no protocol modifications on libraries created using a standard Truseq preparation and fragment size of ~300–400 bp. Each library pool contained eight dual-indexed samples that were pooled prior to capture enrichment. Enriched pools were then sequenced using a Novaseq 6000 with 150 bp paired-end reads. Variants were called from the subsequent data following the protocols outlined in Joynson *et al.* (2021). The resulting single nucleotide polymorphisms (SNPs) for each panel member were combined and utilized for population genetics analysis; after filtering for <10% missing data and >5% minor allele frequency, 269 390 SNPs were retained. To determine genetic similarity between lines, SNPs were subjected to principal component analysis (PCA) carried out in Python using Scikit learn, and the first two eigenvectors were plotted. Further genetic comparison was made for two lines selected from the field experiment with contrasting phenotypes, but that appeared genetically similar (51 and 64, see further details below). A total of 964 107 genome-wide SNP loci were compared between the two lines to determine genomic regions of similarity and difference. SNPs were placed into 5 Mbp bins of genomic sequence and the number of sites with identity by state between the two lines within each bin was deduced with a custom script written in Python.

### Two contrasting lines: glasshouse conditions—plant material and growth

Based on data from field experiments (Table 1; Supplementary Table S1), two wheat lines that contrasted for *V*_c,max,25(HS)_/*N*_area_ (at tillering, anthesis, and grain-filling stages), GY, and total biomass, but genetically similar (Supplementary Fig. S1), were evaluated in more detail under controlled conditions. Their cross names are TITMOUSE and BCN/WBLL1//PUB94.15.1.12/WBLL1, and here they are referred to as 51 and 64, respectively. Line 64 is a high-yielding line generated by strategic crosses, with a Mexican landrace background (PUB94.15.1.12), Bacanora (BCN, high grain number), and Weebill (Weebill, high grain weight) in its pedigree. Line 51 is a comparatively lower yielding line selected from the systematic screening of 70 000 genetic resources under drought and heat, based on its performance under these conditions. It is a Mexican elite line with the pedigree PI/3/INIA66/CIANO//CAL/4/Bluejay ‘S’ from the 1970s (selection history CM30136-2Y-2M-2Y-0M).

**Table 1.** Physiological traits measured on the flag leaves at booting (Zadoks 4.3–4.5), anthesis (Zadoks 6.5), and grain filling (7 d after anthesis; A+7) using hyperspectral reflectance; and yield traits determined at physiological maturity for the two wheat lines 51 and 64 grown for 2 years in northeast Mexico under fully irrigated conditions as part of the panel photosynthetic tails (PStails)

	Parameter	Line	Line	Student’s *t*-test *P*-value
		51	64	
**Grain filling (A+7)**	GY (g m^–2^)	463 ± 14	612 ± 15	**<0.001**
	GM2 (grains m^–2^)	13 392 ± 343	14 256 ± 369	0.112
	TGW (g)	34.5 ± 0.5	42.9 ± 0.4	**<0.001**
	Total biomass (g m^–2^)	1106 ± 29	1371 ± 41	**0.004**
	HI	0.43 ± 0.01	0.45 ± 0.01	0.073
	*V*_c,max,25(HS)_ (µmol m^–2^ s^–1^)	140 ± 16	156 ± 19	0.657
	*V*_c,max,25(HS)_/*N*_area_ [µmol s^–1^ (g N)^–1^]	56 ± 1	63 ± 1	**0.018**
	*J*_(HS)_ (µmol m^–2^ s^–1^)	202 ± 27	219 ± 31	0.757
	*J*_(HS)_/*N*_area_ [µmol s^–1^ (g N)^–1^]	75 ± 7	84 ± 7	0.504
	*N*_area_ (g m^–2^)	2.6 ± 0.1	2.6 ± 0.2	0.798
	*N*_mass_ (mg g^–1^)	55.5 ± 2.2	57.1 ± 3.4	0.785
	SPAD	49.6 ± 1.3	49.6 ± 2.2	0.989
	LMA (g m^–2^)	50.7 ± 1.1	47.6 ± 1.4	0.222
**Anthesis**	*V*_c,max,25(HS)_ (µmol m^–2^ s^–1^)	102 ± 1	153 ± 22	0.127
	*V*_c,max(HS)_/*N*_area_ [µmol s^–1^ (g N)^–1^]	53 ± 1	65 ± 3	**0.032**
	*J*_(HS)_ (µmol m^–2^ s^–1^)	153 ± 6	221 ± 36	0.208
	*J*_(HS)_/*N*_area_ [µmol s^–1^ (g N)^–1^]	70 ± 4	79 ± 6	0.358
	*N*_area_ (g m^–2^)	2.2 ± 0.1	2.7 ± 0.3	0.208
	*N*_mass_ (mg g^–1^)	45.1 ± 1.4	55.1 ± 3.3	0.079
	SPAD	46.0 ± 0.6	49.7 ± 1.4	0.115
	LMA (g m^–2^)	47.0 ± 1.8	50.0 ± 1.2	0.317
**Initiation of booting**	*V*_c,max,25(HS)_ (µmol m^–2^ s^–1^)	167 ± 5	169 ± 7	0.892
	*V*_c,max(HS)_/*N*_area_ [µmol s^–1^ (g N)^–1^]	68 ± 1	68 ± 1	0.861
	*J*_(HS)_ (µmol m^–2^ s^–1^)	228 ± 10	228 ± 16	0.998
	*J*_(HS)_/*N*_area_ [µmol s^–1^ (g N)^–1^]	88 ± 3	88 ± 4	0.975
	*N*_area_ (g m^–2^)	2.6 ± 0.1	2.6 ± 0.1	0.978
	*N*_mass_ (mg g^–1^)	53.0 ± 1.9	52.3 ± 3.1	0.886
	SPAD	47.8 ± 0.9	49.0 ± 1.2	0.856
	LMA (g m^–2^)	52.7 ± 2.2	53.0 ± 3.1	0.958

Values are means ±SEM (*n*=4, i.e. two biological replicates per year).

Seeds of lines 51 and 64 were sown in 3 litre pots containing commercial compost mix (Petersfield Growing Medium, Leicester, UK). Twelve replicate plants of each line were grown in a glasshouse at 26/18 °C day/night with a photoperiod of 16 h. Natural light was supplemented with high pressure sodium lamps (SON-T 400 W, Philips Lighting, Eindhoven, The Netherlands) when external light was lower than 200 W m^–2^. When in use, the supplementary lights provide a minimum of ~500 µmol m^–2^ s^–1^, measured at canopy level using a LI-190R sensor (LI-COR). Pots, each containing one plant, were distributed randomly in the glasshouse, and watered daily to field capacity. Line 51 shows faster development; therefore, seeds from line 64 were sown 12 d before those of line 51, so that plants of the two lines reached booting (Zadoks stage 4.5) and were measured at the same time.

### Two contrasting lines: glasshouse conditions—photosynthetic CO _**2**_**responses and leaf sampling**

Two LI-6800F portable IRGA systems (software version 1.3.17, LI-COR) were used to assess photosynthetic parameters in the two wheat genotypes. Response curves of *A*_CO2_ to the intercellular CO_2_ concentration (*c*_i_) combined with the quantum yield of PSII, Φ_PSII_ (*F*_m_ʹ–*F*_t_/*F*_m_ʹ) from chlorophyll fluorescence (using a multiphase flash) were measured in the mid-section of the flag leaf when the plants reached Zadoks stage 4.3–4.5. For all measurements, leaf temperature was maintained at 25 °C, VPD_leaf_ at ~1.3 kPa, PAR of 1500 µmol m^–2^ s^–1^, and flow rate of 500 µmol s^–1^. Leaves were enclosed in the cuvette and induced to steady state at 43 Pa CO_2__r; under this CO_2__r a CO_2_ concentration in the sample analyser (CO_2__s) of 40.6 ± 2.8 Pa was obtained, close to the current 41 Pa atmospheric concentration (NOAA, https://gml.noaa.gov/ccgg/trends/). CO_2__r was then stepped down through 35, 27, 20, 15, and 5 Pa, and increased to 43, 48, 53, 58, 63, 68, 73, 79, 85, and 95 Pa. Before the data for each step were logged, the reference and sample gas analyser signals were matched. The minimum and maximum wait time for stability were 60 s and 120 s, respectively.

The response of *A*_CO2_ to *c*_i_ was modelled as described by Taylor *et al.* (2020), but using temperature-dependent constants derived for wheat (Silva-Pérez *et al*., 2017; Supplementary Table S2). The relationship between *A*_CO2_ and [CO_2_] was described using a version of the FvCB model (Farquhar *et al.*, 1980; von Caemmerer and Farquhar, 1981) with a simple function for limitation by triose phosphate utilization (*T*_p_; Sharkey *et al.*, 2007). The approach of Gu *et al.* (2010) was used, where all possible carboxylation limitation–state combinations were tested, given the required order of limitation states along the *c*_i_ axis (Rubisco limited <electron transport limited <*T*_p_ limited) and the minimum number of data necessary for each limitation state (*n* ≥2 when Michaelis constants for Rubisco catalysis of carboxylation, *K*_C_, and oxygenation reactions*, K*_O_; and the photosynthetic CO_2_ compensation point in the absence of mitochondrial respiration in the light, Γ*, are fixed). The R Language and Environment function optim (R Core Team, 2018) was used to minimize the distribution-wise cost function, and the model with the lowest cost function value was accepted after checking for admissibility and, if necessary, testing for co-limited ‘swinging points’ (Gu *et al.*, 2010).

Mean leaf temperatures measured in the LI-6800F were used to predict Γ*, *K*_C_, and *K*_O_, using values for wheat (Silva-Pérez *et al*., 2017; Supplementary Table S2). We compared three alternative parameterizations for mesophyl conductance (*g*_m_): *g*_m_ ~∞ (approximated by setting *g*_m_ to 1 × 10^6^ μmol m^−2^ s^−1^ Pa^−1^); *g*_m_=5.5 μmol m^−2^ s^−1^ Pa^−1^, consistent with Silva-Pérez *et al*. (2017); and estimation of *g*_m_ from the data. Of these, only *g*_m_ ~∞ both credibly predicted limitation states indicated by Φ_PSII_ (e.g. Busch and Sage, 2017) and usually led to fitted values of day respiration (*R*_day_) >0. Values for *V*_c,max,25(*A*/*c*i)_, *J*_(*A*/*c*i)_, and *T*_p_ are thus apparent rates that may underestimate true values obtained with a finite estimate of *g*_m_. Similarly, while the CO_2_ compensation point, Γ, is a close match for the data, and *c*_i_ transitions marking boundaries between *A*_C_, *A*_J_, and *A*_P_ were broadly consistent with trends in Φ_PSII_, they depend on the value assigned to *g*_m._

Stomatal limitation (*L*_s_) was calculated from the *A*_CO2_/*c*_i_ curve (Farquhar and Sharkey, 1982). An example of a fitted *A*_CO2_/*c*_i_ response curve and the different parameters derived from it can be seen in Supplementary Fig. S2. Intrinsic water use efficiency (iWUE) was calculated as *A*_CO2_/*g*_s_.

After the *A*_CO2_/*c*_i_ response curve, leaves were acclimated back to steady state at 43 Pa CO_2__r. Once steady state was reached, a sample incorporating the leaf lamina surface inside the cuvette was freeze-clamped within 10 s of opening the chamber (rapidly cooled to the boiling point of liquid N_2_). Measurement of leaf width of the frozen sample and the width of any gap between the leaf edge and the perimeter of the freeze-clamp tongs enabled precise calculation of the sampled area (Carmo-Silva *et al.*, 2017). Samples were stored at –80 °C until extraction.

### Two contrasting lines: glasshouse conditions—biochemistry

Leaf homogenates were extracted from the samples (3.1 cm^2^ total area) previously harvested and stored at –80 °C by grinding the leaves at 4 °C with an ice-cold pestle and mortar containing 0.8 ml of extraction buffer (according to Carmo-Silva *et al.*, 2017 with slight modifications, as described in Sales *et al.*, 2020). The homogenate was clarified by centrifugation at 14 000 *g* and 4 °C for 1 min, and the supernatant was immediately used for measuring Rubisco activity at 25 °C, by incorporation of ^14^CO_2_ into acid-stable products, according to Parry *et al.* (1997) and as detailed in Sales *et al.* (2020). Initial and total Rubisco activities were determined, and the activation state was calculated from the ratio of initial and total activities.

Rubisco and total soluble protein contents were determined in the same supernatant, by the [^14^C]CABP (carboxyarabinitol bisphosphate) binding assay (Whitney *et al.*, 1999) and Bradford method (Bradford, 1976) with BSA as standard, respectively.

Chlorophyll determination followed the method described by Wintermans and de Mots (1965). A 20 µl aliquot of homogenate was taken before centrifugation and added to 480 µl of ethanol, mixed by inversion, and kept in the dark for at least 4 h. After centrifugation, chlorophyll content was determined by the absorbance at 649 nm and 665 nm, using a microplate reader (SPECTROstar Nano, BMG LabTeck, Aylesbury, UK).

A leaf sample adjacent to the region used for gas exchange was collected, oven-dried at 70 °C, and ground to a fine powder using a ball mill (Retsch MM400, Retsch UK Limited, Castleford, UK). Subsamples containing 6–8 mg of leaf powder were wrapped into tin capsules and analysed for carbon and nitrogen (%) using an elemental analyser (VARIO Micro Cube, Hanau, Germany).

### PStails panel: glasshouse conditions—plant material and growth

Addressing the unexpected lack of correspondence between phenotypic properties displayed by the two contrasting genotypes under field versus glasshouse environment conditions, data were analysed for the 80 lines that make up the PStails panel, plus the UK modern spring wheat cultivar Paragon, grown in glasshouse conditions for detailed phenotyping. The ambient conditions in the glasshouses were the same as described in the section ‘Two contrasting lines: glasshouse conditions—plant material and growth’. Four replicates were used, with one plant of each genotype represented in each of four replicate blocks. Due to space constraints, two blocks were grown at the same time in one glasshouse while the other two blocks were planted 17 d later in a second glasshouse set to the same environmental conditions. Maximum and minimum temperature in the two glasshouses during the experimental period are shown in Fig. 1E. Solar radiation measured with an LP02 pyranometer (Campbell Scientific, Logan, UT, USA) by the closest weather station to the experimental location (http://es-websupp.lancs.ac.uk/hazelrigg/) is shown in Fig. 1F.

Plants were grown in 3 litre pots containing commercial compost mix (Petersfield Growing Medium, Leicester, UK). Plants within each block were distributed according to a random design using the Edgar II Experimental Design Generator and Randomiser (Brown, 2005), and were watered daily to field capacity.

### PStails panel: glasshouse conditions—photosynthetic measurements

Three LI-6400XT portable IRGA systems (LI-COR) were used to assess photosynthetic parameters in the wheat genotypes. Response curves of *A*_CO2_ to *c*_i_ were performed in the mid-section of the flag leaf when the plants reached Zadoks stage 4.3–4.5. In all measurements, leaf temperature was maintained at 25 °C, VPD_leaf_ at ~1.3 kPa, PAR of 1500 µmol m^–2^ s^–1^, and a flow rate of 200–300 µmol s^–1^. Leaves were enclosed in the cuvette and induced to steady state at 40 Pa CO_2__r. CO_2__r was then stepped down through 30, 20, 10, and 7 Pa, and increased to 40, 45, 55, 70, 100, and 120 Pa. After the *A*_CO2_/*c*_i_ response curve, leaves were acclimated back to steady state at 40 Pa CO_2__r and PAR of 1800 µmol m^–2^ s^–1^; then PAR was stepped down through 1500, 1000, 500, 250, 120, 50, and 25 µmol m^–2^ s^–1^. Before data for each step were logged, the reference and sample gas analyser signals were matched; the minimum and maximum wait time for stability were 60 s and 120 s, respectively.

*A*_CO2_ measured in the light response curves at PAR of 500 µmol m^–2^ s^–1^ is referred to as the operational photosynthetic rate (*A*_op_)—at similar ambient light to the ambient growth conditions; and at PAR 1800 µmol m^–2^ s^–1^ as *A*_sat_— saturating light. *A*_CO2_/*c*_i_ response curves were fitted according to the photosynthesis of the FvCB model (Farquhar *et al.*, 1980) using the Plantecophys R package (Duursma, 2015), and *V*_c,max,25(*A*/*c*i)_ and *J*_(*A*/*c*i)_ were estimated. *T*_p_ was fitted but data are not presented here as not all lines showed *T*_p_ limitation. Default settings were used for the other parameters.

### PStails panel: glasshouse conditions—phenology and yield components

The time to reach booting (Zadoks stage 4.5) and 50% of anthesis (Zadoks stage 6.5) was recorded for each plant. At the end of the experiment when plants reached physiological maturity (Zadoks stage 8.7), plant height was measured as the length of the main tiller from the soil surface to the tip of the spike excluding the awns. Determination of yield components was conducted using adapted protocols from Pask *et al*. (2012). Each plant was sampled, threshed, oven-dried, and weighed to allow calculation of GY, HI, and biomass at physiological maturity on individual plants. From the harvest of each plant, a subsample of grains was weighed before and after drying (oven-dried to constant weight at 70 °C for 48 h). GY was calculated as grain weight at 85% dry matter, and the ratio of dry grain weight to total dry above-ground biomass was used to determine HI.

### Statistical analyses

For the field work data, adjusted means were calculated for each trait by combining data from the 2 years. Days to heading and days after irrigation were used as the covariate separately (fixed effect) only when its effect was significant (*P*<0.05). For phenology, only days after irrigation was used as a covariate. The ANOVA was conducted with the general linear model (GLM) procedure from META R version 6.01 (Alvarado *et al.*, 2017), with all the effects of years (Y), blocks within replications, replications within years, replications, genotypes (G), and G×Y being considered as random effects.

For the glasshouse experiment with the full PStails panel, the statistical analyses followed the same procedure described above, but the random effects were the different glasshouse (GH) blocks/replications, G, and G×GH. Adjusted means were calculated for each trait using position in the GH as covariate (fixed effect) when its effect was significant. For the gas exchange data, the LI-6400XT (three systems) and time of the day when measurements were performed were used as covariates when their effects were significant.

All figures were prepared in RStudio (version 1.4.1103; RStudio Team, 2021) using the ggplot2 package (Wickham, 2006). For the boxplots comparing lines 51 and 64, outliers were detected and excluded, using the Tukey’s fences method, where outliers are defined as extreme values that are 1.5 times the interquartile range (1.5 IQR) below the first quartile or 1.5 IQR above the third quartile. The Shapiro–Wilk test was performed to evaluate if the data were normally distributed, and the *F*-test was applied to test for homogeneity in the variances of each set of data (for lines 51 and 64). As no significant difference between the variances were found, the parametric *t*-test was applied to test the significance of differences between mean values obtained for each trait for the two lines.

For the linear regressions, Pearson correlation coefficients and probabilities were computed and visualized in RStudio using the packages Hmisc (Harrell, 2019) and corrplot (Wei and Simko, 2017).

## Results

### Two lines with contrasting *V*_c,max,25(HS)_/*N*_area_ traits and similar genetic background under field conditions

Based on 2 years of field experiments with the PStails panel of 80 bread wheat lines (Supplementary Table S1), lines 51 and 64 were selected for detailed characterization in glasshouse conditions as these lines showed contrasting results for high-throughput phenotyping-estimated *V*_c,max,25(HS)_/*N*_area_. Line 51 showed lower *V*_c,max,25_/*N*_area_ at tillering, anthesis, and grain-filling stages, and comparatively lower GY and biomass at physiological maturity than line 64 (Table 1).

To determine the overall level of diversity within the PStails panel, genetic characterization was carried out using PCA (Supplementary Fig. S1A). This analysis split the panel into two main subpopulations across the first eigenvector. To study this similarity in further detail, all genome-wide SNPs for lines 51 and 64 were compared. Overall, ~4.7 Gbp of sequence between the two genotypes were at least 90% similar, represented by ~940 bins of 5 Mbp of genomic sequence across the genome. Chromosomes with the largest regions of similarity (Supplementary Fig. S1B) were 2D where 76% of the chromosome had >90% similarity, followed by 2A (75%), 4A (74%), 1B (72%), 1A (67%), and 3B (50%). The least similar chromosome between the two lines was 7B in which 62% of sequences had an SNP similarity of <20%.

### Detailed analysis of phenotypic traits showed no difference in *V*_c,max,25(*A/ci*)_/*N*_area_ in glasshouse-grown wheat contrasting lines 51 and 64

**Fig. 2.**
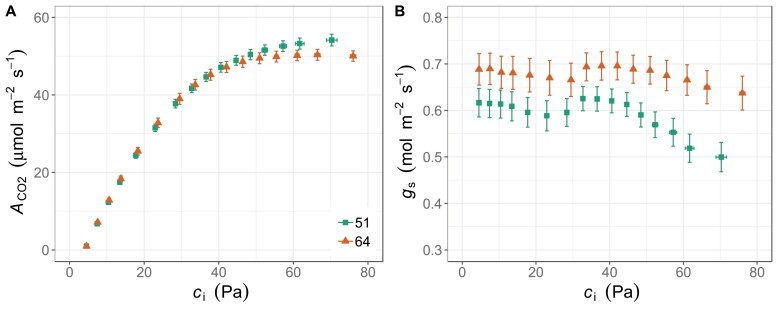
(A) Response curves of net CO_2_ assimilation (*A*_CO2_) and (B) stomatal conductance (*g*_s_) to the intercellular CO_2_ concentration (*c*_i_) in flag leaves of wheat lines 51 and 64 at booting stage grown under glasshouse conditions. Values are means ±SEM (*n*=8–11 biological replicates).

The response of *A*_CO2_ to *c*_i_ for the wheat lines 51 and 64 showed divergence between the two genotypes only at the highest CO_2_ concentrations (Fig. 2A). The genotypes did not differ in *V*_c,max,25(*A*/*c*i)_ and *J*_(*A*/*c*i)_, both corrected for 25 °C (Table 2); however, *J/V*_c,max_ was greater and hence the *c*_i_ at which the limitation of photosynthesis transitions from Rubisco to ribulose bisphosphate (RuBP) regeneration (*c*_i__*c*_J_) occurred at higher *c*_i_ values for line 51 (38.8 ± 0.6 Pa) than for line 64 (34.4 ± 0.9 Pa). For both lines, this transition was above the operating *c*_i_ (i.e. that obtained at the current atmospheric level of 41 Pa and PAR of 500 µmol m^–2^ s^–1^). Furthermore, line 64 showed consistent limitation by *T*_p_, which was not detected in any biological replicates for line 51 (Table 2). The stomatal response to *c*_i_ showed that line 51 had lower stomatal conductance at all *c*_i_ points compared with line 64 (Fig. 2B). This result was consistent with the stomatal limitation (*L*_s_) estimated from the *A*_CO2_/*c*_i_ response curve, higher for line 51 than for line 64 (Table 2). Due to the lower *g*_s_, the iWUE in line 54 was higher than in line 64 when *c*_i_ became higher than 35 Pa (Supplementary Fig. S3).

**Table 2.** Parameters estimated from the response curves of net CO_2_ assimilation (*A*_CO2_) to the intercellular CO_2_ concentration (*c*_i_) in the flag leaves of wheat lines 51 and 64 at booting stage grown under glasshouse conditions

Parameter	Line		Student’s *t*-test *P* value
	51	64	
*V*_c,max,25(*A*/*c*i)_ (μmol m^–2^ s^–1^)	136 ± 4	139 ± 5	0.671
*V*_c,max,25(*A*/*c*i)_/*N*_area_ [µmol s^–1^ (g N)^–1^]	54 ± 2	62 ± 2	0.123
*J*_(*A*/*c*i)_ (μmol m^–2^ s^–1^)	255 ± 7	247 ± 7	0.419
*J*_(*A*/*c*i)_/*N*_area_ [µmol s^–1^ (g N)^–1^]	102 ± 5	109 ± 3	0.392
*c*_i__*c*_J_ (Pa)	38.7 ± 0.7	34.4 ± 0.9	**0.001**
*J/V*_c,max_	1.87 ± 0.01	1.78 ± 0.02	**0.002**
*c*_i___JP_ (Pa)	NA	55.9 ± 1.9	NA
*T*_p_ (μmol m^–2^ s^–1^)	NA	17.0 ± 0.4	NA
Operating *c*_i_ (Pa)	28.1 ± 0.3	29.2 ± 0.3	**0.032**
*R*_d_ (μmol m^–2^ s^–1^)	0.42 ± 0.08	0.46 ± 0.13	0.790
*L*_s_	0.22 ± 0.01	0.17 ± 0.01	**0.004**

Values are means ±SEM (*n*=8–11 biological replicates). *V*_c,max,25(*A*/*c*i)_/*N*_area_ was calculated using N data from Supplementary Fig. S4 (*n*=5–6 biological replicates). *c*_i_JP_ is the *c*_i_ at which the limitation of photosynthesis transitions from from RuBP regeneration to TPU.

**Fig. 3.**
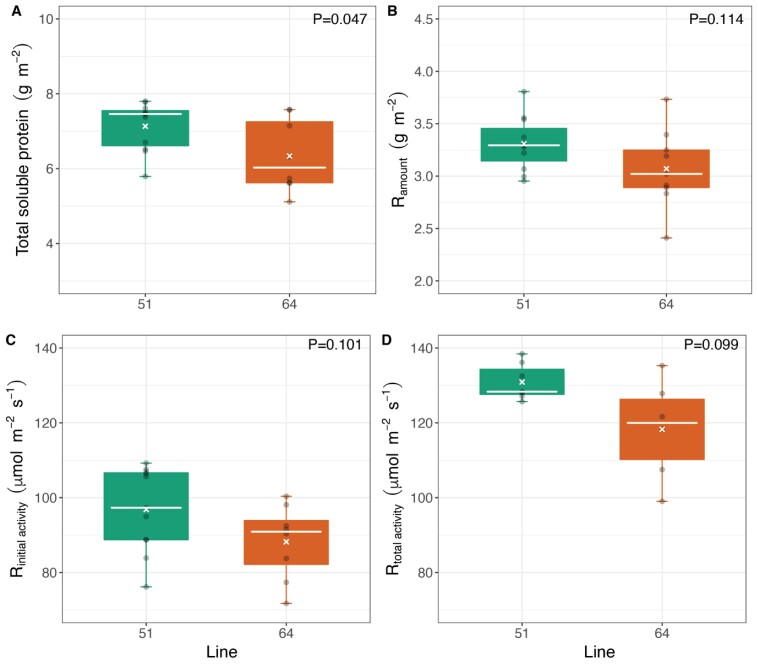
(A) Total soluble protein, (B) Rubisco amounts, and (C) Rubisco initial and (D) total activities in flag leaves of wheat lines 51 and 64 sampled at booting stage. Leaves were sampled after the *A*_CO2_/*c*_i_ response curves, at steady state (PAR of 1500 µmol m^–2^ s^–1^ and 43 Pa CO_2__r). Boxplots show median (white line), mean (white x), interquartile range (IQR, box upper and lower edges), 1.5 times the IQR (whiskers), and individual data points (grey dots). Student’s *t*-test *P*-value is shown for each parameter. *n*=8–10 biological replicates.

Line 51 had a 13% greater N content per unit leaf area compared with line 64 (Supplementary Fig. S4). These results were consistent with the total soluble protein amounts in the leaves (Fig. 3A), with line 51 investing more resources into greater amounts of protein than line 64. Rubisco amounts and activities did not differ significantly between lines (Fig. 3B–D), while Chl *a*, *b*, total chlorophyll, and carotenoid contents were ~24% greater in line 51 (*P*<0.001) than in line 64 (Supplementary Table S3).

Considering that the main parameter used to select lines 51 and 64 from the field experiment was the difference in GY and *V*_c,max,25_/*N*_area_ (estimated through hyperspectral reflectance), *in vivo* and *in vitro* parameters were normalized to N content in the leaves, in order to understand variation in N use efficiency between the lines with contrasting yield. No significant differences were found in *V*_c,max,25(*A*/*c*i)_ (Table 2), Rubisco initial and total activities, and Rubisco amounts between the lines when normalized by N content (Supplementary Fig. S5). On the other hand, total chlorophyll/*N*_area_ and carotenoids/*N*_area_ were significantly higher in the line 51 than in line 64, consistent with results expressed per leaf area (Supplementary Table S3).

### Natural variation in photosynthetic traits amongst the PStails wheat panel grown under glasshouse conditions

**Fig. 4.**
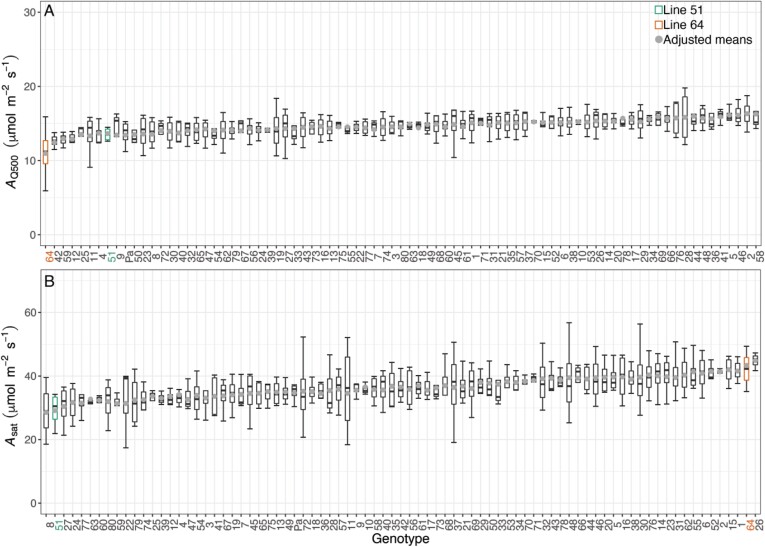
Net CO_2_ assimilation rates at booting stage of flag leaves at 40 Pa CO_2_ and PAR of 500 µmol m^–2^ s^–1^ (*A*_Q500_ or *A*_op_; A) or 1800 µmol m^–2^ s^–1^ (*A*_sat_; B) in the 80 lines of the photosynthetic tails (PStails) panel plus the UK modern spring wheat cultivar cv. Paragon, grown under glasshouse conditions. *A*_CO2_ was measured during the light–response curves. Cultivars are ranked according to the increasing mean of each parameter. Boxplots show median, interquartile range (IQR, box upper and lower edges), and 1.5 times the IQR (whiskers). Grey dots are the adjusted means for *n*=3–4 experimental repetitions. Lines 51 and 64 are highlighted in green and orange, respectively.

The lack of significant differences in Rubisco activity between the two wheat lines (Fig. 3; Table 2) was further supported by phenotyping of photosynthetic traits across the full PStails panel in glasshouse conditions. The rate of *A*_CO2_ measured at ambient CO_2_ and the irradiance experienced by plants in the greenhouse (*A*_Q500_) represent a close approximation to the operational photosynthetic rates (*A*_op_). No significant phenotypic variation in *A*_Q500_ (*P*=0.429) or *A*_sat_ (*P=*0.669) was observed within the PStails lines (Fig. 4).

**Fig. 5.**
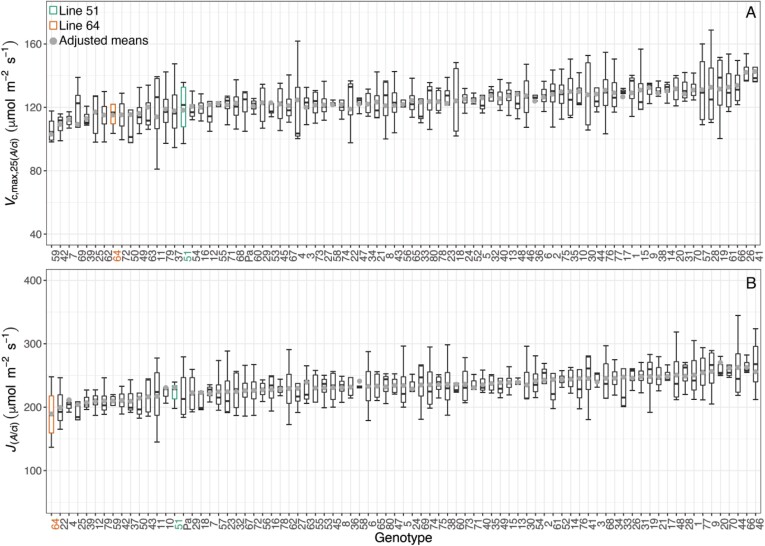
(A) Maximum carboxylation activity of Rubisco [*V*_c,max,25(*A*/*c*i)_] and (B) electron transport rate [*J*_(*A*/*c*i)_] estimated from the response curves of net CO_2_ assimilation (*A*_CO2_) to the intercellular CO_2_ concentration (*c*_i_) in the flag leaves of the 80 lines of the photosynthetic tails (PStails) panel plus the UK modern spring wheat cultivar cv. Paragon, grown under glasshouse conditions. Cultivars are ranked according to increasing mean of each parameter. Boxplots show median, interquartile range (IQR, box upper and lower edges), and 1.5 times the IQR (whiskers). Grey dots are the adjusted means for *n*=3–4 experimental repetitions. Lines 51 and 64 are highlighted in green and orange, respectively.

*V*_c,max,25(*A*/*c*i)_ and *J*_(*A*/*c*i)_, both determined from the *A*_CO2_/*c*_i_ response curves (Fig. 5), did not differ significantly among glasshouse-grown plants of the different lines (*P*=0.884 and *P=*0.380, respectively). The parameters *A*_sat_, *V*_c,max,25(HS)_, and *J*_(HS)_ described above were plotted for the field experiment (Supplementary Fig. S6) to show how the results compared between field and glasshouse experiment. These results were obtained at the booting stage and, while *A*_sat_ was measured using an IRGA system, *V*_c,max,25(HS)_ and *J*_(HS)_ were estimated using hyperspectral reflectance. Again, no significant phenotypic variation was found in *V*_c,max,25(HS)_ (*P=*0.719) or *J*_(HS)_ (*P=*0.480). On the other hand, *A*_sat_ was significantly different between the lines (*P*<0.001) and generally lower for the field-grown than the glasshouse-grown plants.

### HI correlated with *V*_c,max,25_ under field conditions but the correlation shifted to *J* under glasshouse conditions

**Fig. 6.**
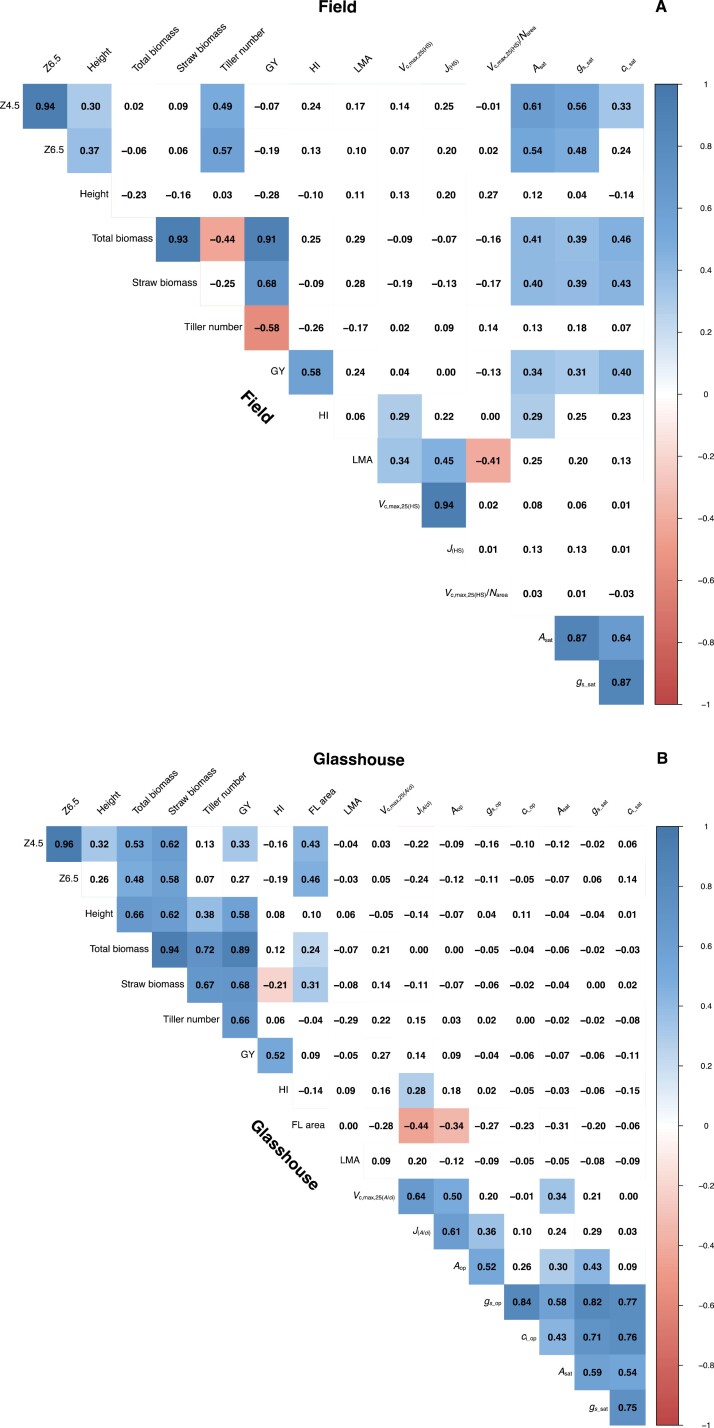

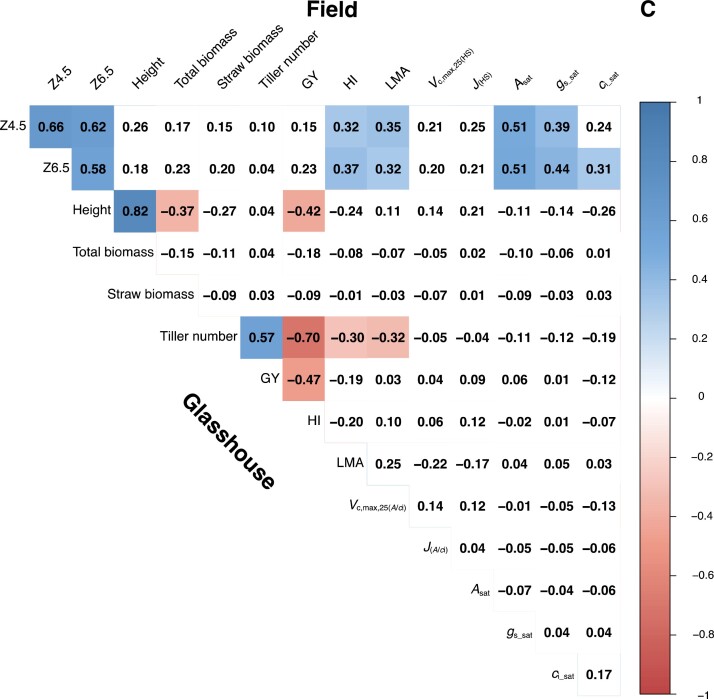
Correlation matrices showing the significance of linear correlation between paired mean values among traits in (A) the field and (B) the glasshouse experiments; and (C) between the two experiments for the 80 lines of the photosynthetic tails (PStails) panel. Numbers are Pearson product–moment correlation coefficients, and increasingly significant correlations are indicated by increasingly darker shading.

Figure 6 shows the correlation matrices between parameters measured under field (Fig. 6A) and glasshouse (Fig. 6B) conditions. In the field dataset (Fig. 6A), *A*_sat_ (i.e. *A*_CO2_ measured at a PAR of 1800 µmol m^–2^ s^–1^) and *V*_c,max,25(HS)_ were positively correlated with HI, whilst under glasshouse conditions (Fig. 6B) only the photosynthetic parameter *J*_(*A*/*c*i)_ correlated with HI, consistent with electron transport limiting photosynthesis at lower irradiance.

Total above-ground biomass correlated strongly and positively with GY in both environments (*r*=0.91 in the field and *r*=0.87 in the glasshouse), and GY also correlated with straw biomass (*r*=0.68). Interestingly, time to reach booting (Zadoks 4.5) and anthesis (Zadoks 6.5) did not correlate with yield parameters in field-grown plants, but showed positive correlation with GY, total above-ground biomass, and straw biomass in the glasshouse-grown plants. While leaf mass per area (LMA) correlated with *V*_c,max,25_, *J*, and *V*_c,max,25_/*N*_area_ under field conditions, this leaf trait did not correlate with any photosynthetic parameter under glasshouse conditions. While different methods were used in the different environments, these results suggest a different set of limitations to plant productivity in glasshouse and field conditions.

### The environment experienced by plants during growth strongly impacts photosynthetic traits

We investigated whether results from glasshouse conditions represented a robust assessment of potential performance under field conditions. The correlations between the values measured across the full PStails panel grown under field versus glasshouse conditions for the different agronomic, photosynthetic, and yield traits are shown in Fig. 6C. The results obtained from glasshouse-grown plants translated well to the field for the agronomic traits (Zadoks stage and height) and GY. However, photosynthetic traits did not show significant correlation between the two experimental conditions.

## Discussion

The initial objective of this study was to identify lines within the PStails panel with contrasting photosynthetic traits but similar genetic background, with the aim of using these lines to generate a double haploid population to further identify markers associated with these photosynthetic traits. Such a population would serve as a resource to identify segregation for multiple traits including *V*_c,max_, biomass production, and Rubisco activity. Using results obtained from 2 years of field experiment, two lines, here called 51 (low tail) and 64 (high tail), were selected (Supplementary Table S1). Although the two genotypes showed a similar genetic background (Supplementary Fig. S1), line 51 had lower *V*_c,max(HS)_/*N*_area_ (measured at anthesis and the grain-filling stage but not at initiation of booting), total biomass, and GY compared with line 64 (Table 1; Supplementary Table S1). When the two genotypes were characterized as part of the PStail panel at booting stage in the glasshouse, results were not consistent with some of the findings under field conditions. In the glasshouse environment, both lines showed low GY and low total biomass compared with the whole panel; the yield advantage of line 64 under field conditions (Supplementary Table S1) was lost in the glasshouse environment (Supplementary Table S4). There was some indication for a difference in *V*_c,max(*A*/*c*i)_/*N*_area_ between genotypes measured under glasshouse conditions, although this was not significant (*P=*0.123), and the absolute values were similar to those obtained in the field experiments at anthesis and grain-filling stages (Tables 1, 2). Overall, our findings highlight the influence of growth environment on the physiological characteristics of wheat and suggest caution when assessing genetic yield potential and variation in photosynthetic traits to inform strategies for crop improvement.

While the detailed characterization of the two lines 51 and 64 under glasshouse conditions did not reveal significant differences between them in *V*_c,max,25(*A*/*c*i)_/*N*_area_ (Table 2), some other differences were detected. For both lines, this transition was above the operating *c*_i_ (i.e. that obtained at the current atmospheric level of 41 Pa and PAR of 500 µmol m^–2^ s^–1^), suggesting that Rubisco activity was limiting photosynthesis in the glasshouse-grown plants. Limitation by *T*_p_ was identified in line 64 at *c*_i_ as low as 49 Pa, but no such effects were found for *c*_i_ values as high as 70 Pa in line 51. The leaves of line 51 had greater N (Supplementary Fig. S4), chlorophyll content (Supplementary Table S3), and iWUE (Supplementary Fig. S3), especially at high *c*_i_, than line 64. Another clear difference was that the operating *c*_i_ was lower for line 51 and, consistent with this, *L*_s_ was greater in line 51 than in line 64 (Table 2). It is interesting to note that *V*_c,max,25(*A*/*c*i)_/*N*_area_, which showed similar absolute values between field (Table 1) and glasshouse (Table 2) experiments, is associated with a shift in *L*_s_ and operating *c*_i_. In addition, the *J*:*V*_c,max_ ratio was significantly greater for genotype 51 than 64, which results in a higher *c*_i_ for the transition from Rubisco- to electron transport-limited *A*_CO2_.

It is well known that Rubisco capacity and photosynthetic rate are highly correlated and, therefore, estimation of modelled parameters reflecting Rubisco capacity (*V*_c,max_) is essential to evaluate photosynthetic performance across different elite crops germplasm (von Caemmerer, 2000; Furbank *et al.*, 2020). *V*_c,max_ combined with photosynthetic electron transport capacity (*J*), another modelled parameter, are more robust than single-point *A*_CO2_ measurements to assess photosynthetic performance in C_3_ plants as they are independent of diurnal variation in *g*_s_ (von Caemmerer, 2000; Condon *et al.*, 2004; Feng *et al.*, 2018; Silva-Pérez *et al*., 2020). When screening for photosynthetic capacity, it is not desirable that the measured parameters vary much due to diurnal changes in the surrounding environment (e.g. soil water availability or light) as it can lead to an underestimation of potential photosynthesis (Condon *et al.*, 2004; Silva-Pérez *et al*., 2020). Furthermore, these parameters have recently been incorporated into a modelling tool that connects leaf-level photosynthesis to crop yield, and highlighted that increases in *V*_c,max_ and *J* increase the simulated wheat yields (Wu *et al.*, 2019). Existing genotypic variation in *V*_c,max_ and *J*, therefore, should be exploited in breeding programmes aiming to improve wheat yield.

The number of studies exploring natural variation in *V*_c,max_ and *J* in wheat has been increasing (Driever *et al.*, 2014; Jahan *et al.*, 2014; Carmo-Silva *et al.*, 2017). However, these parameters are frequently derived from measuring the response of *A*_CO2_ to *c*_i_, which is time-consuming and not easily achievable under field conditions. An alternative method using a leaf reflectance technique to estimate *V*_c,max_ and *J* has been well established in many species (Doughty *et al.*, 2011; Serbin *et al.*, 2012; Ainsworth *et al.*, 2014; Yendrek *et al.*, 2017), including wheat (Silva-Pérez *et al*., 2018, 2020; Khan *et al.*, 2021). This method can dramatically increase phenotyping throughput and shows a correlation of ~0.6–0.7 with photosynthetic parameters predicted via gas exchange (Silva-Pérez *et al*., 2018). In the current work, however, *V*_c,max,25_ and *J* estimated via leaf reflectance under field conditions did not correlate with these parameters estimated via gas exchange in the glasshouse experiment (Fig. 6C). This lack of correlation might be due to the different techniques used or the environmental growth conditions, even though parameters such as *V*_c,max,25_ derived from leaf reflectance seem to be unaffected by the leaf temperature at which reflectance is measured, as shown by Khan *et al.* (2021).

The lack of correlation between results obtained with field-grown and glasshouse-grown plants highlights the complexity of comparing results obtained in different environments (Poorter *et al.*, 2016). Many factors may contribute to the observed differences, but some of the most important are light quantity and quality, as well as the growth temperatures. Plants in the field were exposed to a broader temperature range (lower minimum and higher maximum) and higher maximum daily solar radiation compared with glasshouse conditions (Fig. 1). Even though light under controlled conditions fluctuated much less than under field conditions, plants did not experience saturating light, which would strongly affect processes dependent on light, such as photosynthesis (Poorter *et al*., 2013, 2016). Plants grown under glasshouse conditions in the UK are exposed to relatively low light levels, which means that photosynthesis operation under *J* limitation is expected, and limitations by *V*_c,max_ are less frequent. This is highlighted by the evident difference between lines 51 and 64 in nitrogen allocation. Differences in *V*_c,max,25_ were not detected between the lines at any growth stages under field conditions (Table 1) or at booting stage under glasshouse conditions (Table 2). The differences in *V*_c,max,25_ were detected only when normalized by N content. Although both lines had the same amount of N and SPAD under field conditions, line 51 showed significantly higher nitrogen (Supplementary Fig. S4) and chlorophyll contents (Supplementary Table S3) than line 64 under glasshouse conditions. These results indicate that plants optimize nitrogen allocation to pigments under glasshouse conditions, probably as a strategy to acclimate to low irradiance (Evans, 1989), leading to a higher *J*_(*A*/*c*i)_/*N*_area_ in glasshouse conditions (Table 2) than *J*_(HS)_/*N*_area_ in field grown-plants (Table 1).

Another important factor to be considered under field conditions is the higher temperatures and consequently higher VPD_leaf_ than in the glasshouse, and the more dynamic environment, for example air movement. These factors are likely to drive more frequent stomatal limitation and consequently can lead to *V*_c,max_ limitation more frequently than under glasshouse conditions. This is consistent with the relationship between *g*_s_ (Supplementary Fig. S7A) and *c*_i_ (Supplementary Fig. S7B) measured in the plants grown under field versus glasshouse conditions, since plants under field conditions showed, in general, lower *g*_s_ and *c*_i_ than glasshouse-grown plants.

The timing of phenological phases influences crop yield and is sensitive to photoperiod and cumulative temperature (Richards, 1991; Gómez-Macpherson and Richards, 1997). The number of days to reach anthesis (Zadoks stage 6.5) was significantly correlated between field and glasshouse experiments (*r*=0.57; Supplementary Fig. S8), but the crop cycle was shorter in the glasshouse than in the field. While, for reasons of repeatability, environmental settings are manipulated to obtain a reasonable degree of constancy throughout the growth cycle in glasshouse experiments, the same is not observed in the field, where seasonal progression is a natural complement to progress through phenological stages. In Mexico, temperature and solar radiation were lower at the beginning of the field trial and increased during the crop cycle (Fig. 1). Such increases in photoperiod and temperature should be considered in experiments under glasshouse conditions that aim to assess crop yield for specific environments.

It is noteworthy that under glasshouse conditions, plants were growing individually in pots, which contrasts with the higher plant density experienced under field conditions. In wheat, the number of tillers per plant is strongly affected by sowing density (Lloveras *et al.*, 2004), and genetic variation for tillering capacity has been reported (Fischer *et al.*, 2019). The relationship between tiller number in plants grown in field and glasshouse environments (Supplementary Fig. S9) shows that lines 51 and 64 did not differ in the number of tillers per m^2^ measured in the field but under glasshouse conditions line 64 produced significantly more tillers per plant (11 ± 3) than line 51 (6 ± 2). Plasticity in ear number affects grain yield (Sadras and Rebetzke, 2013) and could contribute to explaining the differences observed between the two growing environments. Plant density can have a range of effects in above- and below-ground responses (Wang *et al.*, 2021). Plant growth in large containers under glasshouse conditions may be an accessible alternative to translate yield results between field and glasshouse experiments. Hohmann *et al.* (2016) have shown high accuracy in predicting yield in oilseed rape using this technique. Use of similar sowing densities to those recommended in the field, and reduced constraints on root development in these large containers, led to above-ground architecture similar to that of field-grown plants. Studies with other crops comparing the impact of pot size on plant physiology and yield (Poorter *et al.*, 2012) would be useful to inform future studies aiming to assess natural variation in photosynthetic traits.

Improving photosynthesis offers untapped potential to increase crop yields (Long *et al.*, 2006; Zhu *et al.*, 2010; Parry *et al.*, 2011; Simkin *et al.*, 2019). With the increasing number of experiments under controlled conditions, as part of efforts to identify genetic variation in photosynthesis for crop yield improvement, the findings presented here suggest caution in designing experiments so that the environmental conditions are closely aligned with the conditions experienced by plants in their target environment and throughout the growth cycle. Field trials complemented with enhanced phenotyping methods under controlled conditions is one of the best approaches to produce reliable data for breeders (Byrne *et al*., 2022). However, not all researchers have access to the field and/or high-throughput phenotyping platforms. Alternative solutions to bridge the gap between field and glasshouse/controlled conditions experiments include higher grade growth cabinets and glasshouses that can be programmed simulating environmental fluctuations experienced by plants under field conditions. However, these types of technologies are not broadly accessible due to their high costs. Furthermore, light intensities in plant growth facilities rarely reach the same level experienced by plants grown under field conditions in the tropics, which can be an obstacle (reviewed by Poorter *et al.*, 2016), especially for crops such as wheat, where the light response saturates at fairly high light intensities above those achieved by most growth cabinets.

Another approach with increasing application in plant sciences is the integration of machine learning with high-throughput phenotyping. Machine learning enables the search for patterns in large datasets containing multiple traits, instead of analysing each factor individually (Ma *et al.*, 2014; Singh *et al.*, 2016). Recent examples of studies combining plant phenotyping with machine learning to predict photosynthetic traits in tobacco (Fu *et al.*, 2019) and wheat (Furbank *et al.*, 2021) showed that this approach improved prediction of photosynthetic traits from leaf hyperspectral reflectance. However, it is important to keep in mind that these studies are dependent on large datasets and high-throughput techniques. Furthermore, the complexity of the machine learning concepts requires expert knowledge for accurate interpretation of results (Ma *et al.*, 2014).

The complex interplay of traits determining crop productivity in dynamic environments experienced by field-grown plants (reviewed by Murchie *et al.*, 2018) should be considered when designing strategies for effective improvement of wheat crop yields. Our findings suggest that when breeding for particular environments, an improved match between phenotypes in field and glasshouse environments will be achieved when experiments are designed so that key conditions are aligned with the cropping cycle in the target breeding environment.

## Supplementary data

The following supplementary data are available at *JXB* online.

Table S1. Summary of 2 years of field experiment results.

Table S2. Kinetic constants used for *V*_c,max,25(*A*/*c*i)_ estimation.

Table S3. Chlorophyll and carotenoid contents for lines 51 and 64.

Table S4. Summary of glasshouse experiment results.

Fig. S1. PCA for the PStails and SNP distribution.

Fig. S2. Example of a fitted *A*_CO2_/*c*_i_ response curve.

Fig. S3. iWUE for lines 51 and 64.

Fig. S4. Carbon and nitrogen content for lines 51 and 64.

Fig. S5. Rubisco parameters normalized to N content for lines 51 and 64.

Fig. S6. *A*_sat_, *V*_c,max,25(HS)_, and *J*_(HS)_ in the PStails panel grown under field conditions.

Fig. S7. *g*_s_ and *c*_i_ relationships between glasshouse- and field-grown plants.

Fig. S8. Relationships between time to reach Zadoks stage 6.5 in glasshouse- and field-grown plants.

Fig. S9. Relationships between the number of tillers in glasshouse- and field-grown plants.

erac096_suppl_Supplementary_Tables_S1-S4_Figures_S1-S9

## Data Availability

The data presented in this publication are available at the data repository used by Lancaster University (https://doi.org/10.17635/lancaster/researchdata/516)

## Abbreviations

*A*_CO2_: net CO_2_ assimilation rate
*A*_op_: operational *A*_CO2_
*A*_sat_: *A*_CO2_ under saturating light
*c*_i_: intercellular CO_2_ concentration
CO_2__r: air CO_2_ concentration in the reference IRGA
*g*_m_: mesophyll conductance
GM2: grains per square metre
*g*_s_: stomatal conductance
*g*_s_op_: *g*_s_ at PAR of 500 μmol m^−2^ s^−1^ and 41 Pa CO_2_
*g*_s_ *_*_sat_: *g*_s_ at PAR of 1800 μmol m^−2^ s^−1^ and 41 Pa CO_2_
GY: grain yield
HI: harvest index
IRGA: infrared gas analyser
*J*_(*A*/*c*i)_: electron transport rate estimated by *A*_CO2_/*c*_i_ curve fitting
*J*_(HS)_: electron transport rate estimated by hyperspectral reflectance
*K*_C_: Michaelis–Menten constant for Rubisco in relation to CO_2_
*K*_O_: Michaelis–Menten constant for Rubisco in relation to O_2_
LMA: leaf mass per area
*L*_s_: stomatal limitation
*N*_area_: leaf nitrogen content per unit leaf area
*N*_mass_: leaf nitrogen content per unit dry mass
*R*_day_: daytime rate of respiration
PAR: photosynthetic active radiation
TGW: thousand grain weight
*T*_p_: triose phosphate utilization rate
*V*_c,max,25(*A*/*c*i)_: *in vivo* maximum carboxylation rate of Rubisco estimated by *A*_CO2_/*c*_i_ curve fitting
*V*_c,max,25(HS)_: *in vivo* maximum carboxylation rate of Rubisco estimated by hyperspectral reflectance
*V*_c;max,25_/*N*_area_: *V*_c,max_ per unit leaf nitrogen
VPD_leaf_: leaf to air vapour pressure difference
Γ*: photosynthetic CO_2_ compensation point in the absence of mitochondrial respiration in the light
Ф_PSII_: quantum yield of PSII
