# Supplementary material for: Phenotypic variation in photosynthetic traits in wheat grown under field versus glasshouse conditions
Source: J Exp Bot. 2022 Mar 10;73(10):3221–37. doi: 10.1093/jxb/erac096 (PMC9126738; doi:10.1093/jxb/erac096)
Supplement: erac096_suppl_Supplementary_Tables_S1-S4_Figures_S1-S9 [file erac096_suppl_supplementary_tables_s1-s4_figures_s1-s9.pdf]

Phenotypic variation in photosynthetic traits in wheat grown under field *versus* glasshouse conditions

Cristina R. G. Sales, Gemma Molero, John R. Evans, Samuel H. Taylor, Ryan Joynson, Robert T. Furbank, Anthony Hall, Elizabete Carmo-Silva

Supplementary data

Table S1. Summary of two years field experiment results.
Table S2. Kinetic constants used for $V_{c,max(A/ci)}$ estimation.
Table S3. Chlorophyll and carotenoid contents for lines 51 and 64.
Table S4. Summary of glasshouse experiment results.
Fig. S1. PCA for the PS tails and SNPs distribution.
Fig. S2. Example of a fitted A_{CO2}/c_i response curve.
Fig. S3. iWUE for lines 51 and 64.
Fig. S4. Carbon and nitrogen content for lines 51 and 64.
Fig. S5. Rubisco parameters normalised to N content for lines 51 and 64.
Fig. S6. A_{sat}, $V_{c,max,25(HS)}$, and $J_{(HS)}$ in the PStails panel grown under field conditions.
Fig. S7. g_s and c_i relationships between glasshouse and field grown plants.
Fig. S8. Relationships between time to reach Zadoks stage 6.5 in glasshouse and field grown plants.
Fig. S9. Relationships between the number of tillers in glasshouse and field grown plants.

Table S1. Adjusted means (*n*=4, i.e., 2 biological replicates per year) of the field experiment results for two years (2016-2017 and 2017-2018) in the 80 wheat lines of the Photosynthetic tails (PStails) panel. Lines selected for more detailed analysis under glasshouse conditions are highlighted.

GENOTYPE ID	CROSS NAME	GID	GY	HI	Total biomass	Vc,max,25(HS)/Narea Tillering	Vc,max,25(HS)/Narea Booting	Vc,max,25(HS)/Narea Anthesis	Vc,max,25(HS)/Narea Grain Filling
1	PAVON F 76	2465	→ 479	→ 0.44	→ 1079	↓ 88.5	↓ 66.5	↓ 55.4	→ 59.1
2	SERI M 82	3895	↑ 554	→ 0.42	↑ 1293	→ 92.0	→ 68.3	↓ 53.5	→ 59.5
3	BACANORA T 88	16122	↑ 571	↑ 0.49	→ 1157	↓ 88.5	→ 69.7	↓ 54.6	→ 59.5
4	ATTILA	41948	↑ 579	↑ 0.46	↑ 1254	→ 90.1	→ 69.3	↓ 51.7	→ 57.8
5	BAVIACORA M 92	447647	↑ 575	↑ 0.45	↑ 1267	→ 92.7	→ 69.0	↓ 57.0	→ 60.6
6	SERI/RAYON	371007	↑ 577	↑ 0.47	→ 1222	→ 90.5	→ 67.0	↓ 54.8	→ 57.7
7	BRBT1*2/KIRITATI	5397958	↑ 596	↑ 0.48	↑ 1244	↓ 85.2	→ 67.0	↓ 51.8	↓ 55.8
8	KRICHAUFF	2447427	→ 501	↓ 0.39	↑ 1315	↓ 86.9	↑ 73.2	↓ 53.6	↓ 54.2
9	SAUAL/WHEAR//SAUAL	6178783	↑ 568	→ 0.44	↑ 1315	→ 94.3	↓ 65.9	→ 64.4	→ 59.5
10	UP2338*2/4/SNI/TRAP#1/3/KAUZ*2/TRAP//KAUZ/5/MILAN/KAUZ//CHIL/CHUM18/6/UP2338*2/4/SNI/TRAP#1/3/KAUZ*2/TRAP//KAUZ	6176178	↑ 579	↑ 0.45	↑ 1300	→ 90.1	→ 71.5	→ 62.1	↓ 52.5
11	PASTOR//HXL7573/2*BAU/3/WBLL1	6676541	↑ 587	↑ 0.45	↑ 1347	→ 92.1	→ 69.3	↓ 57.5	→ 61.2
12	SOKOLL/WBLL1	5429336	↑ 564	↑ 0.47	→ 1207	→ 92.1	→ 69.5	↓ 54.4	→ 60.6
13	MILAN/KAUZ//DHARWAR DRY/3/BAV92	4577931	↑ 591	↑ 0.45	↑ 1308	↑ 95.9	↑ 77.1	↑ 66.2	↑ 62.5
14	W15.92/4/PASTOR//HXL7573/2*BAU/3/WBLL1	5435924	↑ 538	↑ 0.45	→ 1192	→ 91.2	↑ 73.9	→ 58.1	↑ 62.6
15	KUKRI	6154391	↑ 586	↑ 0.48	→ 1237	→ 95.2	↑ 77.0	↑ 71.7	↑ 64.4
16	MUNAL #1	5398530	↑ 529	↑ 0.46	→ 1159	→ 93.4	→ 71.3	↑ 69.1	↑ 63.0
17	PBW343*2/KUKUNA*2//FRTL/PIFED	6179222	↑ 584	↑ 0.48	→ 1227	→ 95.2	→ 68.2	↓ 56.9	→ 58.0
18	JANZ	6062414	→ 505	↑ 0.47	→ 1047	↑ 99.3	→ 69.7	→ 62.8	→ 60.2
19	PI 220463	308163	↓ 314	↓ 0.39	↓ 813	↑ 96.7	↑ 73.6	→ 58.4	→ 58.5
20	EGA BONNIE ROCK	5638672	→ 431	↓ 0.37	→ 1194	→ 90.8	→ 68.5	↓ 53.6	→ 57.7
21	MTRWA92.161/PRINIA/5/SERI*3//RL6010/4*YR/3/PASTOR/4/BAV92	5894989	↑ 522	→ 0.40	↑ 1301	↓ 87.5	→ 69.8	↓ 54.8	→ 59.7
22	SOKOLL	(blank)	↑ 543	→ 0.43	↑ 1250	→ 92.7	→ 67.5	↓ 54.7	→ 56.7
23	PFAU/WEAVER*2//TRANSFER#12,P88.272.2	5397748	↑ 579	↑ 0.47	↑ 1249	→ 92.0	↓ 63.8	↓ 54.7	↓ 56.3
24	C80.1/3*QT4118//KAUZ/RAYON/3/2*TRCH/7/CMH79A.955/4/AGA/3/4*SN64/CNO67//INIA66/5/NAC/6/RIALTO	6489912	↑ 523	↓ 0.40	↑ 1356	↑ 97.8	↑ 73.0	↑ 66.7	↑ 63.1
25	BCN/WBLL1//PUB94.15.1.12/WBLL1	7129702	↑ 588	→ 0.43	↑ 1386	↑ 96.2	→ 68.5	→ 58.5	→ 58.1
26	WBLL1*2/4/SNI/TRAP#1/3/KAUZ*2/TRAP//KAUZ/5/KACHU #1	5995532	↑ 560	→ 0.43	↑ 1308	→ 91.0	↓ 66.5	↓ 54.6	→ 60.1
27	CHEWINK #1	5551750	↑ 555	↑ 0.45	↑ 1259	→ 92.4	↓ 66.4	→ 62.4	↓ 55.6
28	WBLL4//OAX93.24.35/WBLL1/5/CROC_1/AE.SQUARROSA (205)//BORL95/3/PRL/SARA//TSI/VEE#5/4/FRET2	6692366	↑ 607	↑ 0.46	↑ 1339	→ 91.3	→ 69.5	→ 58.1	→ 60.8
29	MEX94.2.19//SOKOLL/WBLL1	6056158	↑ 561	↑ 0.47	→ 1185	→ 94.7	→ 69.4	→ 61.5	→ 58.2
30	WBLL1*2/KURUKU	6056049	↑ 562	↑ 0.46	→ 1220	↑ 98.8	↓ 66.8	↓ 53.6	→ 60.9
31	DPW 621-50	7025958	↑ 565	→ 0.43	↑ 1330	↑ 99.7	→ 67.7	↓ 54.5	→ 56.6
32	MEX94.27.1.20/3/SOKOLL//ATTILA/3*BCN/4/PUB94.15.1.12/WBLL1	7034038	↑ 633	↑ 0.45	↑ 1466	↑ 98.0	→ 70.4	→ 59.0	→ 61.4
33	SOKOLL//PUB94.15.1.12/WBLL1	7129720	↑ 585	→ 0.44	↑ 1328	→ 89.3	→ 68.2	↓ 56.9	→ 58.4
34	ATTILA/3*BCN	1099708	↑ 591	↑ 0.46	↑ 1295	→ 94.9	→ 68.0	→ 60.0	→ 61.3
35	BAV92/SERI	5180627	↑ 557	↑ 0.47	↑ 1241	→ 93.3	→ 70.1	↓ 53.8	→ 59.5
36	PUB94.15.1.12/FRTL/5/CROC_1/AE.SQUARROSA (205)//BORL95/3/PRL/SARA//TSI/VEE#5/4/FRET2	6692380	↑ 572	↑ 0.45	↑ 1278	→ 92.0	→ 70.5	→ 58.9	↑ 65.1
37	BCN/WBLL1//PUB94.15.1.12/WBLL1	7129696	↑ 602	↑ 0.45	↑ 1380	→ 92.5	→ 69.1	↓ 56.7	↑ 65.2
38	KACHU	4755013	↑ 559	→ 0.43	↑ 1289	→ 94.2	→ 67.5	↓ 52.5	→ 57.0
39	C80.1/3*QT4118//KAUZ/RAYON/3/2*TRCH/7/CMH79A.955/4/AGA/3/4*SN64/CNO67//INIA66/5/NAC/6/RIALTO/8/WBLL1*2/KURUKU	6489746	↑ 525	→ 0.40	↑ 1244	→ 92.3	→ 69.9	→ 60.1	↓ 56.0
40	C80.1/3*QT4118//KAUZ/RAYON/3/2*TRCH/4/BERKUT/KRICHAUFF	7129763	↑ 543	→ 0.43	↑ 1289	↑ 96.2	→ 69.4	↑ 67.8	↑ 65.3
41	MEX94.27.1.20/3/SOKOLL//ATTILA/3*BCN	6056170	↑ 579	↑ 0.45	↑ 1305	→ 89.2	→ 69.0	↓ 56.0	→ 60.8
42	MEX94.27.1.20/3/SOKOLL//ATTILA/3*BCN/4/PUB94.15.1.12/WBLL1	7410999	↑ 567	→ 0.44	↑ 1312	↓ 88.0	↑ 73.3	→ 64.3	↑ 62.5
43	WBLL4//OAX93.24.35/WBLL1	6056165	↑ 581	→ 0.44	↑ 1317	↓ 88.9	→ 70.1	↓ 53.9	→ 56.7
44	FRANCOLIN #1/WBLL1	6174859	↑ 582	↑ 0.47	→ 1229	→ 91.1	→ 70.9	→ 57.9	↑ 62.9
45	MUTUS//ND643/2*WBLL1	6683522	↑ 560	↑ 0.48	→ 1166	→ 92.2	→ 67.9	↓ 54.5	→ 57.0
46	QUAIU*2/KINDE	6682171	↑ 619	↑ 0.48	↑ 1315	→ 94.2	→ 70.0	→ 59.6	→ 60.6
47	DANPHE #1*2/CHYAK	6569050	↑ 570	↑ 0.46	→ 1235	↓ 84.8	→ 68.3	↓ 56.3	→ 57.4
48	SUP152*2/TECUE #1	6415924	↑ 588	→ 0.44	↑ 1386	↑ 96.7	→ 70.2	→ 61.8	↓ 56.4
49	CMH79A.955/4/AGA/3/4*SN64/CNO67//INIA66/5/NAC	6062418	↑ 568	→ 0.43	↑ 1276	↓ 85.6	↓ 64.3	↓ 51.9	↓ 55.4
50	BCN/RIALTO//ROLF07	6489593	↑ 596	→ 0.41	↑ 1501	→ 91.3	→ 68.7	→ 57.7	↓ 54.3
**51**	**TITMOUSE**	**419778**	**→ 463**	**→ 0.43**	**→ 1107**	**↓ 82.7**	**→ 67.7**	**↓ 53.1**	**↓ 55.8**
52	KS940935.7.1.2/2*PASTOR/4/FRAME//MILAN/KAUZ/3/PASTOR	7142049	↑ 534	↑ 0.46	→ 1183	↓ 82.8	→ 68.7	→ 58.5	↑ 63.5
53	SOKOLL//PBW343*2/KUKUNA/3/ATTILA/PASTOR	6000921	↑ 580	→ 0.43	↑ 1345	→ 90.2	→ 71.9	→ 57.7	→ 61.3
54	PASTOR//HXL7573/2*BAU/3/ATTILA/3*BCN/4/ATTILA/PASTOR	6692262	↑ 596	↑ 0.47	↑ 1279	↓ 82.8	→ 67.2	↓ 55.7	↑ 63.7
55	MASSIV/PPR47.89C	5228230	↑ 519	↑ 0.47	→ 1113	→ 94.1	↑ 74.1	→ 61.4	→ 61.6
56	CHEN/AE.SQ//2*WEAVER/3/BAV92/4/JARU/5/OLI2/SALMEJA/6/CROC_1/AE.SQUARROSA (205)//BORL95/3/PRL/SARA//TSI/VEE#5/4/FRET2	6692412	↑ 578	↑ 0.47	→ 1204	↓ 85.3	→ 71.7	→ 60.4	→ 58.8
57	CROC_1/AE.SQUARROSA (224)//OPATA/3/PUB94.15.1.12/WBLL1	7129751	↑ 570	↑ 0.45	↑ 1254	→ 89.7	→ 69.8	→ 61.6	→ 58.6
58	WBLL4//OAX93.24.35/WBLL1/5/CROC_1/AE.SQUARROSA (205)//BORL95/3/PRL/SARA//TSI/VEE#5/4/FRET2	6692363	↑ 595	↑ 0.45	↑ 1328	→ 94.4	→ 68.3	↓ 54.8	↑ 62.3
59	VORB//PARUS/PASTOR	6384782	↑ 582	→ 0.41	↑ 1464	→ 91.6	→ 67.1	↓ 52.7	→ 58.5
60	D67.2/PARANA 66.270//AE.SQUARROSA (320)/3/CUNNINGHAM	6056055	↑ 547	→ 0.44	→ 1227	→ 93.6	↑ 75.9	→ 64.6	↑ 64.9
61	CMH79A.955/4/AGA/3/4*SN64/CNO67//INIA66/5/NAC/6/RIALTO/7/ROLF07	6489569	→ 501	→ 0.44	→ 1130	↑ 96.7	→ 67.9	↓ 50.6	↓ 51.7
62	ATTILA//PGO/SERI/3/PASTOR	4883021	↑ 574	→ 0.43	↑ 1347	→ 90.6	→ 67.8	↓ 54.6	→ 58.0
63	PANDORA//WBLL1*2/BRAMBLING	6177058	↑ 595	↑ 0.46	↑ 1297	→ 93.5	→ 67.0	↓ 52.5	↓ 56.4
**64**	**BCN/WBLL1//PUB94.15.1.12/WBLL1**	**7129703**	**↑ 612**	**↑ 0.45**	**↑ 1371**	**→ 92.6**	**→ 67.9**	**→ 64.7**	**↑ 62.7**
65	SOKOLL//PUB94.15.1.12/WBLL1	7129732	↑ 554	→ 0.44	↑ 1267	↑ 99.1	→ 71.2	→ 64.7	→ 60.4
66	KUKRI/EXCALIBUR	6154214	↑ 581	↑ 0.46	↑ 1306	→ 94.2	→ 67.7	↓ 54.9	↓ 56.0
67	SOKOLL*2/TROST	6000909	↑ 578	↑ 0.45	↑ 1286	→ 90.8	↓ 62.0	↓ 51.2	↓ 52.0
68	MEX94.2.19/PUB94.15.1.12	6056184	↑ 573	→ 0.40	↑ 1522	↓ 87.2	→ 69.5	→ 59.1	↑ 62.1
69	DRYSDALE	5245560	↑ 573	→ 0.44	↑ 1313	→ 89.6	→ 68.1	↓ 54.6	↑ 62.2
70	JNRB.5/PIFED/5/BJY/COC//PRL/BOW/3/SARA/THB//VEE/4/PIFED	7032458	↑ 617	↑ 0.45	↑ 1383	→ 92.9	→ 67.1	↓ 55.2	↓ 56.3
71	FIRETAIL	66369	→ 487	↑ 0.48	→ 1065	→ 89.6	→ 69.4	↓ 54.5	↓ 55.8
72	JAL95.4.9	221692	→ 490	↑ 0.45	→ 1130	↓ 88.0	→ 69.3	→ 58.7	→ 60.0
73	CHIH95.4.12	5895795	↓ 260	↓ 0.40	↓ 672	↑ 102.3	↑ 73.3	↑ 66.7	↑ 61.9
74	MEX94.28.2	192155	↓ 294	↓ 0.36	↓ 815	↑ 96.3	→ 69.2	→ 60.1	↑ 62.0
75	OAX92.2.5	157648	↓ 325	↓ 0.38	↓ 858	→ 89.8	→ 72.1	→ 61.6	→ 59.5
76	PASTOR//HXL7573/2*BAU/3/MEX94.2.19//ATTILA/3*BCN	6056182	↑ 575	↑ 0.46	↑ 1266	↑ 96.1	↓ 66.8	→ 57.8	→ 60.4
77	OAX93.10.1	5895769	↓ 247	↓ 0.38	↓ 666	↑ 102.2	↑ 72.8	↑ 70.5	↑ 66.7
78	CHIH95.3.29	223822	↓ 271	↓ 0.40	↓ 696	↑ 98.2	↑ 72.3	↑ 68.5	→ 61.0
79	H.OSM	3825359	→ 414	↓ 0.37	→ 1147	↓ 86.8	→ 70.3	↑ 64.9	→ 60.2
80	VEE/MJI//2*TUI/3/2*PASTOR	3822959	↑ 543	→ 0.42	↑ 1296	→ 92.3	→ 68.6	→ 58.8	↓ 56.4

Abbreviations: genotype id, identifier of the lines used in this study; GID, genotypic identifier at CIMMYT; GY, grain yield; HI, harvest index; $V_{c,max25(HS)}/N_{area}$, *in vivo* maximum carboxylation activity of Rubisco (estimated by hyperspectral reflectance) per unit leaf nitrogen.

Table S2. The Michaelis-Menten constants for CO_2 (K_C) and O_2 (K_O), and photorespiratory compensation point (Γ^*) used to fit A_{CO2}/c_i response curves for the experiments performed with the two contrasting wheat lines 51 and 64 under glasshouse conditions.

Kinetic constant	at 25°C	E_a (kJ mol^{-1})
K_C	27.2 Pa	93.72
K_O	16.6 kPa	33.6
Γ^*	3.774 Pa	24.42

Temperature corrected values were obtained after Silva-Perez *et al.*, 2017*, using $P = P_{25} e^{\left(\frac{E_a(T-25)}{R \cdot 298 \cdot (T+273.15)}\right)}$.

***Silva-Pérez V, Furbank RT, Condon AG, Evans JR. 2017.** Biochemical model of C_3 photosynthesis applied to wheat at different temperatures. Plant, Cell and Environment, **40,** 1552–1564.

Table S3. Chlorophyll and carotenoid contents, and their values normalised by the amount of nitrogen in the flag leaves of the two wheat lines 51 and 64 at booting stage grown under glasshouse conditions.

	**Line**		**Student's *t*-test**
**Parameter**	**51**	**64**	**P value**
Chl *a* (mg m^{-2})	495.3 ± 14.1	409.1 ± 15.3	**0.001**
Chl *b* (mg m^{-2})	196.7± 5.6	164.1 ± 5.2	**<0.001**
Chl *a*/*b*	2.52 ± 0.01	2.49 ± 0.03	0.359
Total Chl (mg m^{-2})	692.0 ± 19.7	573.2 ± 20.3	**0.001**
Total Chl/*N* [mg (g N)$^{-1}$]	285.2 ± 7.6	242.6 ± 10.5	**0.048**
Carotenoids (mg m^{-2})	113.7 ± 3.0	92.9 ± 3.6	**<0.001**
Carotenoids/N [mg (g N)$^{-1}$]	46.1 ± 1.4	38.7 ± 1.6	**0.044**

Values are means ± SEM (*n* = 8-11 biological replicates). Values normalised by N content were calculated using N data from Fig. S4 (*n* = 5-6 biological replicates).

Table S4. Adjusted means (*n*=3-4) of the glasshouse conditions experiment results for the 80 wheat lines of the Photosynthetic tails (PStails) panel plus the UK modern spring wheat cultivar Paragon. Lines selected from the field results (Table S1) for more detailed analysis under glasshouse conditions are highlighted.

GENOTYPE ID	CROSS NAME	GID	GY	HI	Total biomass	Vc,max(A/ci)	J(A/ci)
1	PAVON F 76	2465	↓ 11.0	→ 0.40	↓ 24	→ 129	↑ 251
2	SERI M 82	3895	→ 16.5	→ 0.41	→ 35	→ 128	→ 243
3	BACANORA T 88	16122	→ 11.4	→ 0.40	↓ 19	→ 120	→ 240
4	ATTILA	41948	↓ 9.1	↓ 0.27	→ 27	→ 125	↓ 212
5	BAVIACORA M 92	447647	→ 16.8	→ 0.39	→ 36	→ 126	→ 235
6	SERI/RAYON	371007	↓ 10.5	→ 0.40	↓ 21	→ 127	→ 233
7	BRBT1*2/KIRITATI	5397958	↓ 8.2	→ 0.35	↓ 20	↓ 112	→ 222
8	KRICHAUFF	2447427	→ 14.2	↑ 0.46	→ 26	→ 121	→ 231
9	SAUAL/WHEAR//SAUAL	6178783	→ 15.4	→ 0.42	→ 32	↑ 133	↑ 257
10	UP2338*2/4/SNI/TRAP#1/3/KAUZ*2/TRAP//KAUZ/5/MILAN/KAUZ//CHIL/CHUM18/6/UP2338*2/4/SNI/TRAP#1/3/KAUZ*2/TRAP//KAUZ	6176178	→ 18.1	→ 0.37	↑ 42	↑ 130	→ 231
11	PASTOR//HXL7573/2*BAU/3/WBLL1	6676541	→ 12.1	↑ 0.48	↓ 22	↓ 114	→ 218
12	SOKOLL/WBLL1	5429336	→ 16.4	↑ 0.46	→ 30	→ 122	↓ 210
13	MILAN/KAUZ//DHARWAR DRY/3/BAV92	4577931	↑ 19.9	→ 0.42	↑ 41	→ 127	→ 240
14	W15.92/4/PASTOR//HXL7573/2*BAU/3/WBLL1	5435924	↓ 10.9	→ 0.36	→ 24	↑ 130	↑ 244
15	KUKRI	6154391	↓ 9.7	→ 0.41	↓ 21	↑ 131	→ 239
16	MUNAL #1	5398530	↓ 8.8	→ 0.33	↓ 23	→ 120	→ 228
17	PBW343*2/KUKUNA*2//FRTL/PIFED	6179222	→ 15.7	↑ 0.47	→ 29	→ 127	↑ 249
18	JANZ	6062414	→ 16.1	↑ 0.46	→ 30	→ 124	→ 224
19	PI 220463	308163	↑ 22.8	↑ 0.45	↑ 44	↑ 132	↑ 249
20	EGA BONNIE ROCK	5638672	→ 17.3	↑ 0.43	→ 33	↑ 132	↑ 271
21	MTRWA92.161/PRINIA/5/SERI*3//RL6010/4*YR/3/PASTOR/4/BAV92	5894989	↓ 8.9	↑ 0.49	↓ 17	→ 124	↑ 248
22	SOKOLL	(blank)	↓ 8.6	→ 0.38	↓ 19	→ 119	↓ 200
23	PFAU/WEAVER*2//TRANSFER#12,P88.272.2	5397748	↓ 11.0	↓ 0.22	→ 38	→ 123	→ 225
24	C80.1/3*QT4118//KAUZ/RAYON/3/2*TRCH/7/CMH79A.955/4/AGA/3/4*SN64/CNO67//INIA66/5/NAC/6/RIALTO	6489912	→ 14.5	→ 0.38	→ 33	→ 126	→ 229
25	BCN/WBLL1//PUB94.15.1.12/WBLL1	7129702	↓ 9.8	→ 0.33	→ 27	→ 117	↓ 204
26	WBLL1*2/4/SNI/TRAP#1/3/KAUZ*2/TRAP//KAUZ/5/KACHU #1	5995532	↑ 22.0	↑ 0.44	↑ 43	↑ 142	↑ 246
27	CHEWINK #1	5551750	↑ 20.3	→ 0.38	↑ 44	→ 122	→ 230
28	WBLL4//OAX93.24.35/WBLL1/5/CROC_1/AE.SQUARROSA (205)//BORL95/3/PRL/SARA//TSI/VEE#5/4/FRET2	6692366	↓ 9.9	↑ 0.45	↓ 19	↑ 133	↑ 249
29	MEX94.2.19//SOKOLL/WBLL1	6056158	→ 12.7	→ 0.38	→ 31	→ 123	→ 223
30	WBLL1*2/KURUKU	6056049	→ 18.0	↑ 0.44	→ 35	→ 128	→ 235
31	DPW 621-50	7025958	↓ 10.3	→ 0.43	↓ 20	→ 129	↑ 249
32	MEX94.27.1.20/3/SOKOLL//ATTILA/3*BCN/4/PUB94.15.1.12/WBLL1	7034038	→ 14.5	→ 0.40	→ 31	→ 128	→ 225
33	SOKOLL//PUB94.15.1.12/WBLL1	7129720	→ 16.3	→ 0.42	→ 33	→ 123	↑ 248
34	ATTILA/3*BCN	1099708	↓ 7.3	→ 0.33	↓ 20	→ 122	↑ 246
35	BAV92/SERI	5180627	↓ 10.4	→ 0.33	→ 27	↑ 130	→ 238
36	PUB94.15.1.12/FRTL/5/CROC_1/AE.SQUARROSA (205)//BORL95/3/PRL/SARA//TSI/VEE#5/4/FRET2	6692380	→ 18.5	↑ 0.48	→ 34	→ 124	→ 232
37	BCN/WBLL1//PUB94.15.1.12/WBLL1	7129696	→ 14.1	→ 0.38	→ 32	→ 118	↓ 210
38	KACHU	4755013	↓ 9.8	↑ 0.45	↓ 19	→ 129	→ 236
39	C80.1/3*QT4118//KAUZ/RAYON/3/2*TRCH/7/CMH79A.955/4/AGA/3/4*SN64/CNO67//INIA66/5/NAC/6/RIALTO/8/WBLL1*2/KURUKU	6489746	→ 17.1	→ 0.31	↑ 46	↓ 113	↓ 208
40	C80.1/3*QT4118//KAUZ/RAYON/3/2*TRCH/4/BERKUT/KRICHAUFF	7129763	→ 11.4	→ 0.30	→ 32	→ 126	→ 236
41	MEX94.27.1.20/3/SOKOLL//ATTILA/3*BCN	6056170	↑ 19.7	↑ 0.47	→ 36	↑ 143	↑ 246
42	MEX94.27.1.20/3/SOKOLL//ATTILA/3*BCN/4/PUB94.15.1.12/WBLL1	7410999	→ 17.7	→ 0.40	→ 39	↓ 109	↓ 211
43	WBLL4//OAX93.24.35/WBLL1	6056165	→ 12.3	→ 0.34	→ 33	→ 121	→ 217
44	FRANCOLIN #1/WBLL1	6174859	→ 11.5	↑ 0.44	↓ 23	→ 129	↑ 263
45	MUTUS//ND643/2*WBLL1	6683522	→ 13.3	↑ 0.45	→ 25	→ 122	→ 231
46	QUAIU*2/KINDE	6682171	→ 18.7	↑ 0.48	→ 33	→ 127	↑ 256
47	DANPHE #1*2/CHYAK	6569050	↓ 10.6	↑ 0.43	↓ 21	→ 122	→ 235
48	SUP152*2/TECUE #1	6415924	→ 12.5	→ 0.43	→ 25	→ 129	↑ 251
49	CMH79A.955/4/AGA/3/4*SN64/CNO67//INIA66/5/NAC	6062418	→ 13.7	↑ 0.46	→ 25	→ 116	→ 239
50	BCN/RIALTO//ROLF07	6489593	→ 12.9	→ 0.32	→ 33	↓ 116	↓ 215
51	TITMOUSE	419778	↓ 4.2	→ 0.42	↓ 9	→ 118	→ 231
52	KS940935.7.1.2/2*PASTOR/4/FRAME//MILAN/KAUZ/3/PASTOR	7142049	↓ 6.2	→ 0.36	↓ 14	→ 124	→ 244
53	SOKOLL//PBW343*2/KUKUNA/3/ATTILA/PASTOR	6000921	→ 16.0	↑ 0.47	→ 30	→ 123	→ 231
54	PASTOR//HXL7573/2*BAU/3/ATTILA/3*BCN/4/ATTILA/PASTOR	6692262	↓ 9.7	→ 0.41	↓ 21	→ 121	→ 242
55	MASSIV/PPR47.89C	5228230	↓ 10.6	↑ 0.46	↓ 20	→ 123	→ 231
56	CHEN/AE.SQ//2*WEAVER/3/BAV92/4/JARU/5/OLI2/SALMEJA/6/CROC_1/AE.SQUARROSA (205)//BORL95/3/PRL/SARA//TSI/VEE#5/4/FRET2	6692412	↓ 4.0	↓ 0.24	↓ 12	→ 122	→ 228
57	CROC_1/AE.SQUARROSA (224)//OPATA/3/PUB94.15.1.12/WBLL1	7129751	↑ 20.4	↑ 0.46	→ 39	↑ 131	→ 225
58	WBLL4//OAX93.24.35/WBLL1/5/CROC_1/AE.SQUARROSA (205)//BORL95/3/PRL/SARA//TSI/VEE#5/4/FRET2	6692363	↓ 3.8	↓ 0.16	↓ 18	→ 122	→ 241
59	VORB//PARUS/PASTOR	6384782	↓ 9.1	↑ 0.45	↓ 18	↓ 103	↓ 210
60	D67.2/PARANA 66.270//AE.SQUARROSA (320)/3/CUNNINGHAM	6056055	↑ 24.5	→ 0.42	↑ 50	→ 121	→ 236
61	CMH79A.955/4/AGA/3/4*SN64/CNO67//INIA66/5/NAC/6/RIALTO/7/ROLF07	6489569	↑ 22.2	↑ 0.49	→ 39	↑ 133	→ 244
62	ATTILA//PGO/SERI/3/PASTOR	4883021	→ 17.5	↑ 0.50	→ 30	↓ 115	→ 230
63	PANDORA//WBLL1*2/BRAMBLING	6177058	↑ 19.7	↑ 0.48	→ 36	→ 120	→ 240
64	BCN/WBLL1//PUB94.15.1.12/WBLL1	7129703	↓ 11.1	→ 0.35	→ 26	↓ 115	↓ 190
65	SOKOLL//PUB94.15.1.12/WBLL1	7129732	→ 11.3	↓ 0.26	→ 37	→ 125	→ 234
66	KUKRI/EXCALIBUR	6154214	→ 11.2	→ 0.37	→ 26	↑ 134	↑ 263
67	SOKOLL*2/TROST	6000909	→ 18.0	→ 0.40	→ 36	→ 121	→ 226
68	MEX94.2.19/PUB94.15.1.12	6056184	→ 14.1	↑ 0.53	↓ 23	→ 123	↑ 246
69	DRYSDALE	5245560	→ 13.6	↑ 0.49	→ 25	↓ 110	→ 235
70	JNRB.5/PIFED/5/BJY/COC//PRL/BOW/3/SARA/THB//VEE/4/PIFED	7032458	→ 14.0	↑ 0.48	→ 25	↑ 131	↑ 262
71	FIRETAIL	66369	↑ 19.3	↑ 0.44	→ 39	→ 122	→ 231
72	JAL95.4.9	221692	↓ 8.8	→ 0.38	↓ 20	↓ 116	→ 227
73	CHIH95.4.12	5895795	↑ 24.8	↑ 0.49	↑ 44	→ 122	→ 237
74	MEX94.28.2	192155	↑ 26.2	→ 0.39	↑ 54	→ 122	→ 236
75	OAX92.2.5	157648	↑ 22.8	↑ 0.47	↑ 42	↑ 130	→ 236
76	PASTOR//HXL7573/2*BAU/3/MEX94.2.19//ATTILA/3*BCN	6056182	↑ 22.0	↑ 0.55	→ 36	↑ 131	↑ 246
77	OAX93.10.1	5895769	↑ 21.5	↑ 0.49	→ 38	→ 129	↑ 255
78	CHIH95.3.29	223822	↑ 23.4	↑ 0.49	↑ 42	→ 124	→ 229
79	H.OSM	3825359	→ 14.5	↑ 0.50	→ 25	→ 119	↓ 210
80	VEE/MJI//2*TUI/3/2*PASTOR	3822959	→ 17.4	↑ 0.47	→ 32	→ 124	→ 232
Pa	Paragon	NA	→ 14.8	↓ 0.23	↑ 51	→ 121	→ 222

Abbreviations: genotype id, identifier of the lines used in this study; GID, genotypic identifier at CIMMYT; GY, grain yield; HI, harvest index; $V_{c,max(A/c_i)}$, *in vivo* maximum carboxylation activity of Rubisco, and $J_{(A/c_i)}$, electron transport rate (estimated by A_{CO_2}/c_i curve fitting).

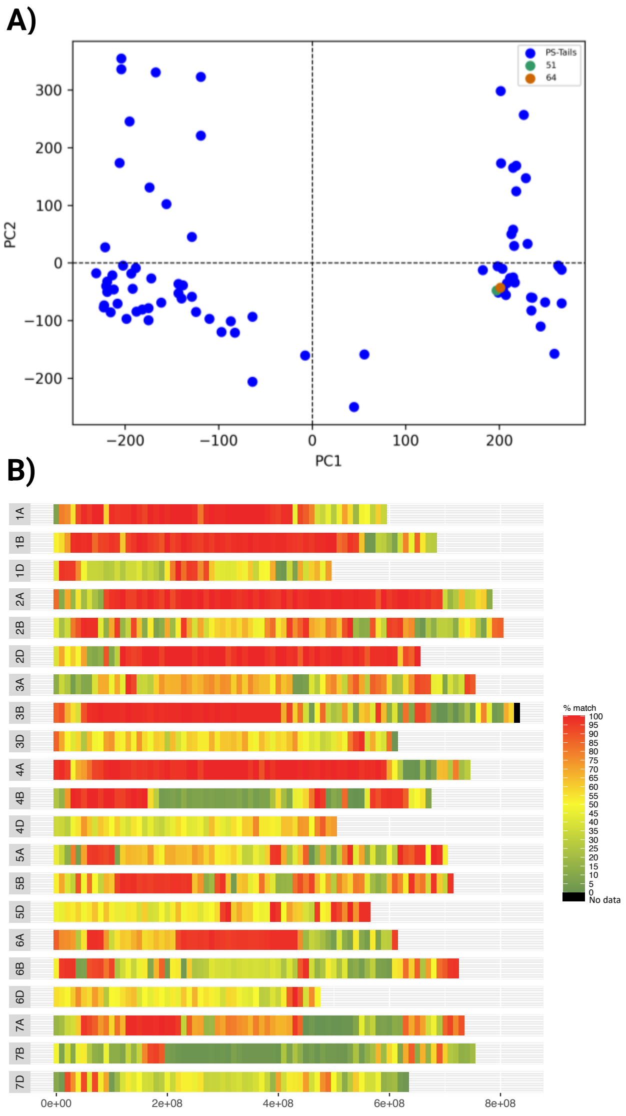

Fig. S1. (A) Principal component analysis (PCA) for the PS tails panel grown under field conditions showing lines 51 and 64 highlighted in green and orange, respectively; and (B) SNPs distribution from enrichment capture data after filtering for <10% missing data and >5% minor allele frequency (MAF).

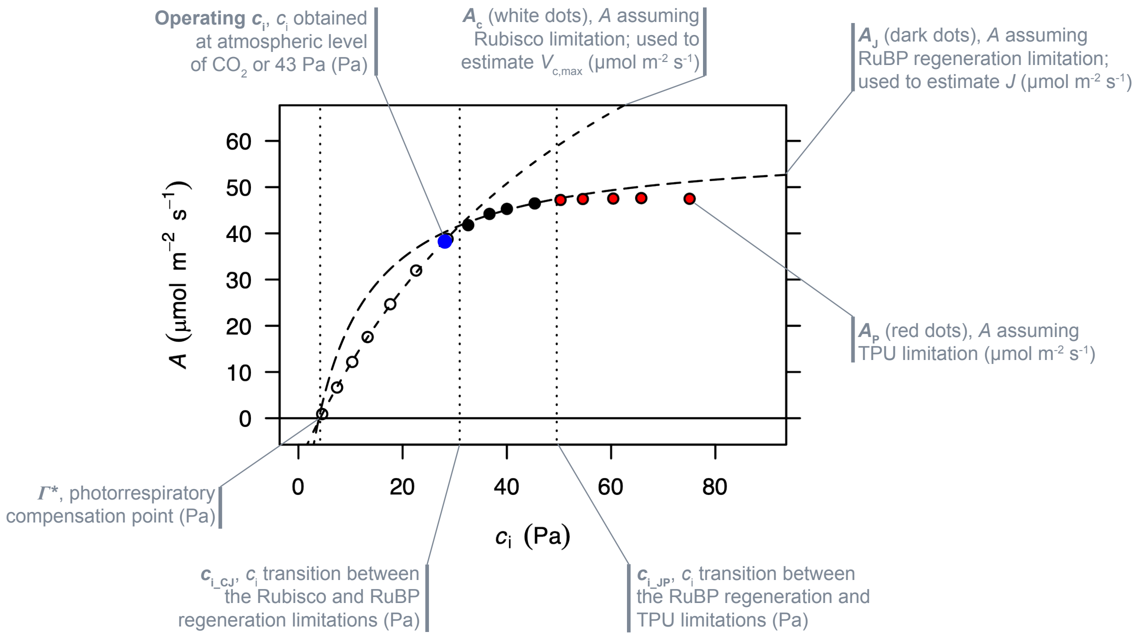

Fig. S2. Example of a fitted response curves of net CO_2 assimilation (A_{CO2}) to the intercellular CO_2 concentration (c_i) and the different parameters derived from it used to compare the two wheat lines 51 and 64 under glasshouse conditions.

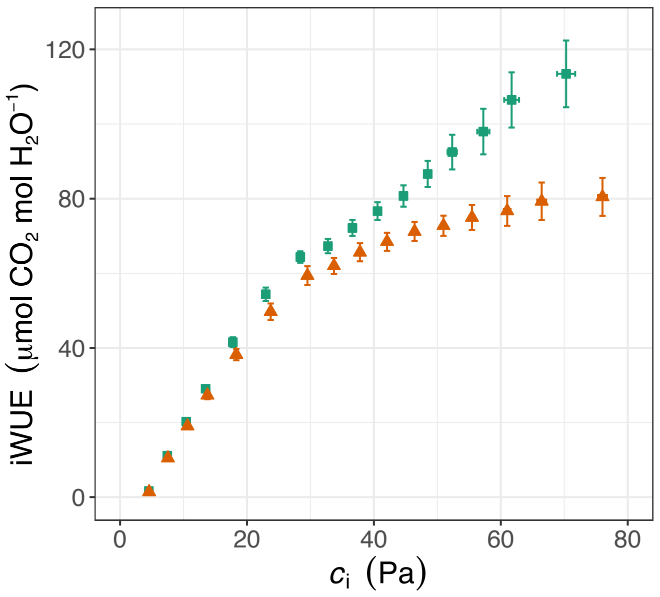

Fig. S3. Intrinsic water use efficiency (iWUE) in flag leaves of wheat lines 51 (green squares) and 64 (orange triangles) at booting stage grown under glasshouse conditions. Values are means ± SEM (n = 8-11).

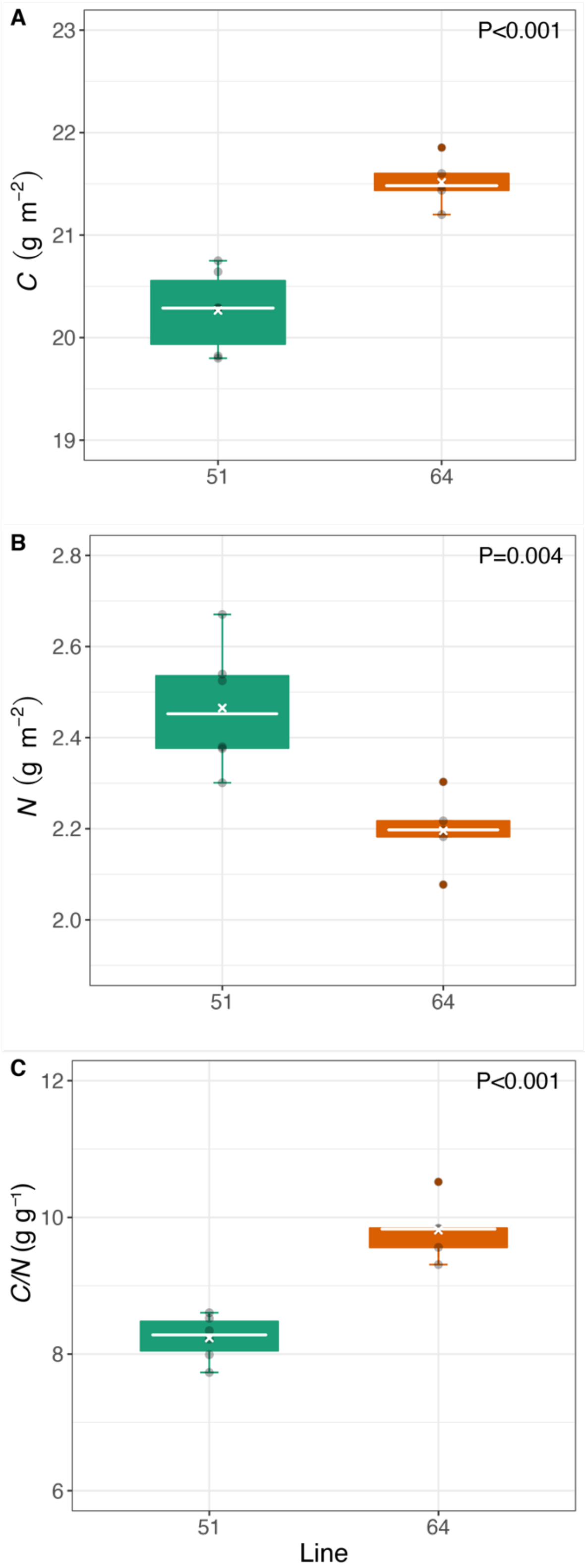

Fig. S4. (A) Carbon (*C*) and (B) nitrogen (*N*) content per unit leaf area, and (C) *C*/*N* ratio in flag leaves of wheat lines 51 and 64 sampled at booting stage, grown under glasshouse conditions. Boxplots show median (white line), mean (white x), inter-quartile range (IQR, box upper and lower edges), 1.5 times of IQR (whiskers) and individual data points (grey dots). Student's *t*-test *P* value is shown for each parameter (n = 5-6).

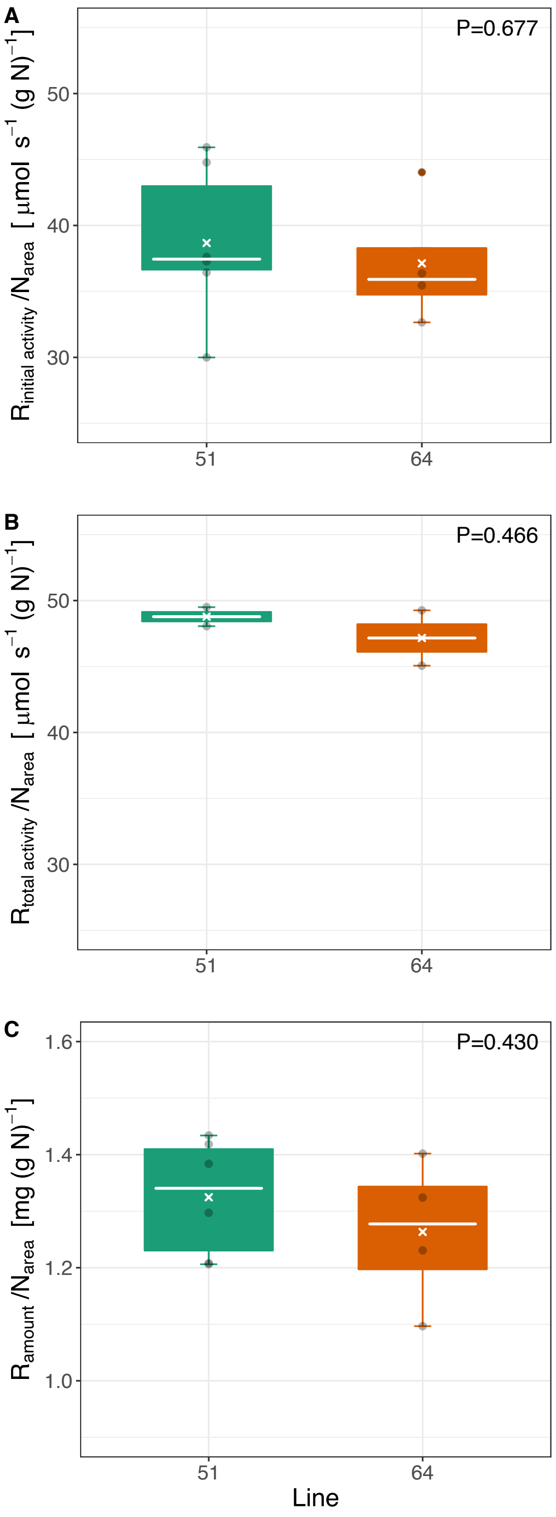

Fig. S5. (A) Rubisco initial and (B) total activities, (C) and Rubisco amounts normalised by the nitrogen content in flag leaves of wheat lines 51 and 64 sampled at booting stage, grown under glasshouse conditions. Leaves were sampled after the A/c_i response curves, at steady state (PAR of 1500 µmol m^{-2} s^{-1} and 43 Pa CO_2_r). Boxplots show median (white line), mean (white x), inter-quartile range (IQR, box upper and lower edges), 1.5 times of IQR (whiskers) and individual data points (grey dots). Student's *t*-test *P* value is shown for each parameter (n = 5-6).

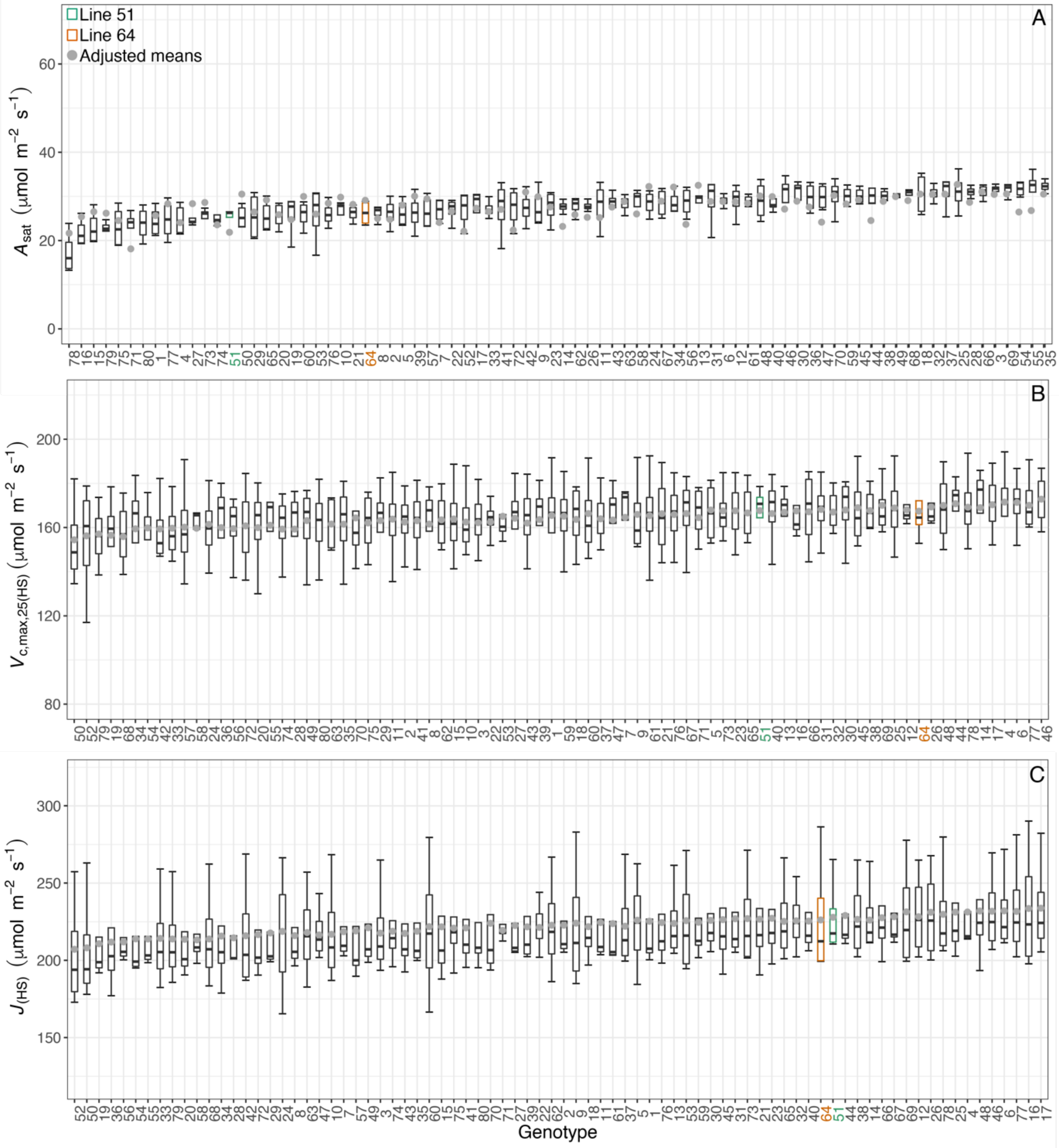

Fig. S6. (A) Net CO_2 assimilation rate at booting stage of flag leaves at PAR of 1800 μmol m^{-2} s^{-1} (A_{sat}), (B) maximum carboxylation activity of Rubisco ($V_{c,max(HS)}$), and (C) electron transport rate ($J_{(HS)}$) in the 80 lines of the photosynthetic tails (PStails) panel grown under field conditions for two years (2016-2017 and 2017-2018). Cultivars are ranked according to increasing mean of each parameter. Boxplots show median, inter-quartile range (IQR, box upper and lower edges), and 1.5 times of IQR (whiskers). Grey dots are the adjusted means of the field experiment results for two years. The lines 51 and 64 are highlighted in green and orange, respectively.

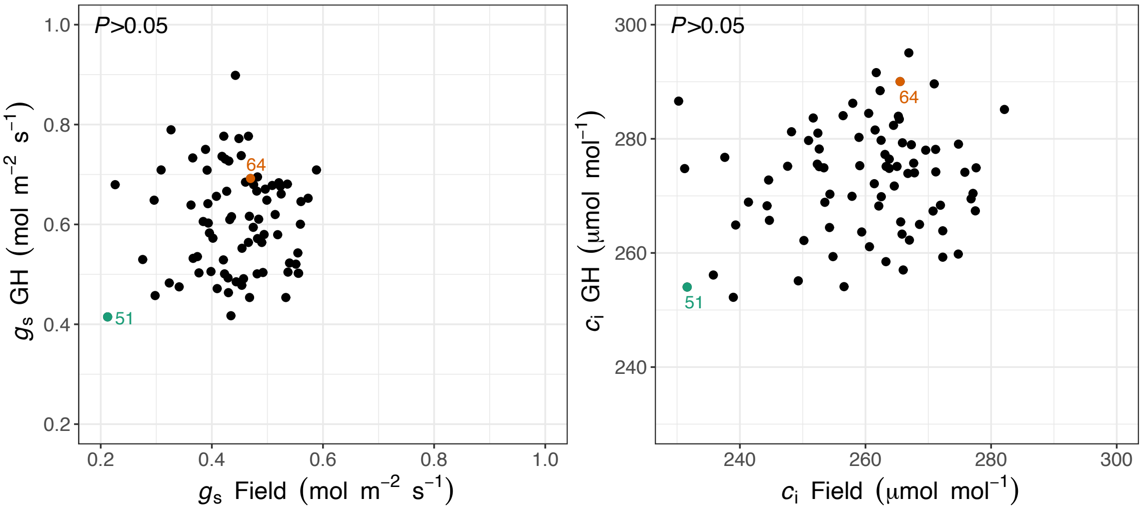

Fig. S7. The relationship between (A) stomatal conductance (g_s) and (B) intercellular CO_2 concentration (c_i) in flag leaves in the 80 wheat lines of the Photosynthetic tails (PStails) panel at booting stage grown under field or glasshouse (GH) conditions. Parameters were measured under PAR of 1800 µmol m^{-2} s^{-1}. Values are adjusted means of two years (2016-2017 and 2017-2018) for the field experiment and of *n*=3-4 experimental repetitions in the GH. The lines 51 and 64 are highlighted in green and orange, respectively.

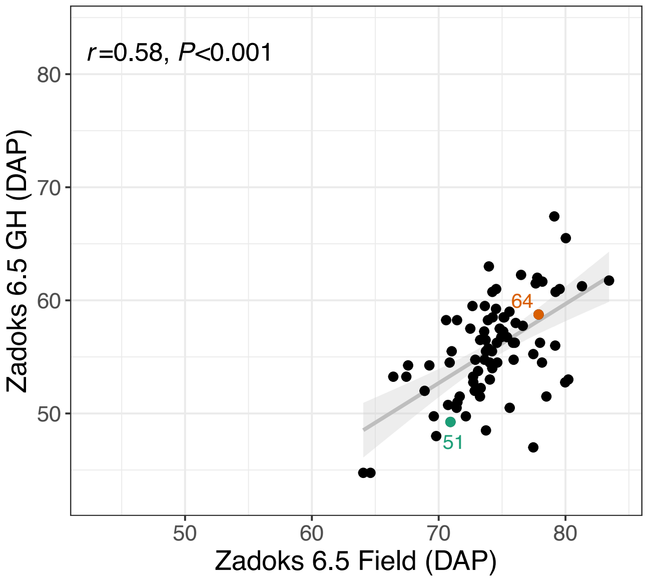

Fig S8. The relationship between the time to reach anthesis (Zadoks 6.5) in the 80 wheat lines of the Photosynthetic tails (PStails) panel grown under field or glasshouse (GH) conditions. Values are adjusted means of two years (2016-2017 and 2017-2018) for the field experiment and of *n*=3-4 experimental repetitions in the GH. The lines 51 and 64 are highlighted in green and orange, respectively.

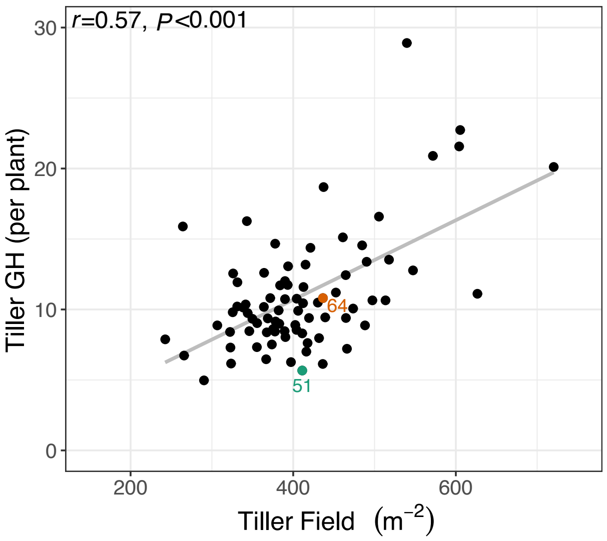

Fig S9. The relationship between the number of tillers in the 80 wheat lines of the Photosynthetic tails (PStails) panel grown under field or glasshouse (GH) conditions. Values are adjusted means of two years (2016-2017 and 2017-2018) for the field experiment and of *n*=3-4 experimental repetitions in the GH. The lines 51 and 64 are highlighted in green and orange, respectively.
